# Role of the androgen receptor in breast cancer and preclinical analysis of enzalutamide

**DOI:** 10.1186/bcr3599

**Published:** 2014-01-22

**Authors:** Dawn R Cochrane, Sebastián Bernales, Britta M Jacobsen, Diana M Cittelly, Erin N Howe, Nicholas C D’Amato, Nicole S Spoelstra, Susan M Edgerton, Annie Jean, Javier Guerrero, Francisco Gómez, Satyanarayana Medicherla, Iván E Alfaro, Emma McCullagh, Paul Jedlicka, Kathleen C Torkko, Ann D Thor, Anthony D Elias, Andrew A Protter, Jennifer K Richer

**Affiliations:** 1 Department of Pathology, University of Colorado Anschutz Medical Campus, RC1 North 5th Floor P18-5127 12800 E. 19th Ave, Aurora, CO 80045, USA; 2 Medivation Inc., 525 Market Street, 36th Floor, San Francisco, CA 94105, USA; 3 Fundación Ciencia & Vida, Avda. Zañartu 1482 - Ñuñoa, Santiago 7780272, Chile; 4 Department of Medicine, Division of Oncology, University of Colorado Anschutz Medical Campus, Aurora, CO, USA

## Abstract

**Introduction:**

The androgen receptor (AR) is widely expressed in breast cancers and has been proposed as a therapeutic target in estrogen receptor alpha (ER) negative breast cancers that retain AR. However, controversy exists regarding the role of AR, particularly in ER + tumors. Enzalutamide, an AR inhibitor that impairs nuclear localization of AR, was used to elucidate the role of AR in preclinical models of ER positive and negative breast cancer.

**Methods:**

We examined nuclear AR to ER protein ratios in primary breast cancers in relation to response to endocrine therapy. The effects of AR inhibition with enzalutamide were examined *in vitro* and in preclinical models of ER positive and negative breast cancer that express AR.

**Results:**

In a cohort of 192 women with ER + breast cancers, a high ratio of AR:ER (≥2.0) indicated an over four fold increased risk for failure while on tamoxifen (HR = 4.43). The AR:ER ratio had an independent effect on risk for failure above ER % staining alone. AR:ER ratio is also an independent predictor of disease-free survival (HR = 4.04, 95% CI: 1.68, 9.69; p = 0.002) and disease specific survival (HR = 2.75, 95% CI: 1.11, 6.86; p = 0.03). Both enzalutamide and bicalutamide inhibited 5-alpha-dihydrotestosterone (DHT)-mediated proliferation of breast cancer lines *in vitro*; however, enzalutamide uniquely inhibited estradiol (E2)-mediated proliferation of ER+/AR + breast cancer cells. In MCF7 xenografts (ER+/AR+) enzalutamide inhibited E2-driven tumor growth as effectively as tamoxifen by decreasing proliferation. Enzalutamide also inhibited DHT- driven tumor growth in both ER positive (MCF7) and negative (MDA-MB-453) xenografts, but did so by increasing apoptosis.

**Conclusions:**

AR to ER ratio may influence breast cancer response to traditional endocrine therapy. Enzalutamide elicits different effects on E2-mediated breast cancer cell proliferation than bicalutamide. This preclinical study supports the initiation of clinical studies evaluating enzalutamide for treatment of AR^+^ tumors regardless of ER status, since it blocks both androgen- and estrogen- mediated tumor growth.

## Introduction

In breast cancers, androgen receptor (AR) is more widely expressed than estrogen receptor alpha (ER) or progesterone receptor (PR), and AR has recently emerged as a useful marker for the further refinement of breast cancer subtype classification [1,2]. Of all 2,171 invasive breast cancers in women enrolled in the Nurses' Health Study, 77% were positive for AR by immunohistochemistry [3]. Among the subtypes, 88% of ER+, 59% of HER2+, and 32% of triple-negative breast cancers (ER–/PR–/HER2–) were positive for AR expression by immunohistochemistry [3]. Similar to ER and PR, AR expression is associated with a well-differentiated state [4] and with more indolent breast cancers [5].

In ER + breast cancers, adjuvant treatment with the competitive antagonist tamoxifen or aromatase inhibitors (AIs), which block conversion of androgens to estrogens, is generally effective for inhibiting disease progression. However, 30 to 50% of all ER + tumors display *de novo* resistance to traditional endocrine therapies and ultimately all metastatic ER + breast cancers acquire resistance [6,7]. In ER + tumors that respond to neoadjuvant endocrine therapy, we previously observed that AR mRNA and protein expression decrease, while in tumors that fail to respond AR mRNA does not decrease [8,9]. AR overexpression increases tamoxifen resistance in breast cancer models *in vitro* and *in vivo*[10]. Thus, *de novo* or acquired resistance to anti-estrogen therapies could result from tumor cell adaptation from estrogen dependence to androgen dependence. In mice, treatment with an AI markedly elevated intratumoral testosterone concentrations in dimethylbenz(*a*)anthracene-induced rat mammary tumors [11]. In postmenopausal women with ER + breast cancer, particularly those being treated with AIs, circulating levels of estradiol (E2) are extremely low, while circulating androgen levels are increased [12] since AIs block the conversion of androgens to estrogen. Indeed, circulating levels of testosterone, androstenedione, and dehydroepiandrosterone sulfate (DHEA-S) increase in women on AI therapy [13] as compared with pretreatment levels. Furthermore, high levels of the adrenal androgen DHEA-S before treatment are predictive of failure on AIs, and circulating DHEA-S increased during treatment in patients with tumors that failed to respond to AI treatment [14].

A subset of ER– breast cancers (molecular apocrine or luminal androgen receptor) retain AR [15-18] and have a gene expression pattern that closely resembles that of ER + breast cancers [2,19]. The anti-androgen bicalutamide inhibits the growth of molecular apocrine cell lines *in vitro* and *in vivo*, supporting the hypothesis that anti-androgens may be useful targeted therapies for such tumors [2,17,18,20]. Indeed, a phase II clinical trial testing bicalutamide as treatment for ER–/AR + breast cancers (NCT00468715) showed some efficacy [21]. Bicalutamide is a competitive antagonist that does allow AR to bind DNA [22]; however, in the setting of castrate-resistant prostate cancer, bicalutamide can exhibit an antagonist-to-agonist shift as demonstrated clinically by a decline in prostate-specific antigen following bicalutamide (Casodex) withdrawal [23].

Enzalutamide (formerly MDV3100) is an AR signaling inhibitor that binds AR with fivefold higher affinity than bicalutamide, impairs AR nuclear translocation, and lacks agonist activity at effective doses [20-23]. Enzalutamide significantly improved overall survival in patients with castrate-resistant prostate cancer and is an approved agent for the treatment of patients with metastatic castration-resistant prostate cancer [24]. In this study, we examined the effect of enzalutamide in AR + breast cancer models (ER + and ER–) and present the first preclinical evidence that inhibition of AR with enzalutamide may be an effective therapeutic strategy not only for ER–/AR + breast cancers, but also for ER+/AR + tumors. We also present clinical data which suggest that a high amount of AR relative to ER may be indicative of tumors that will have a less than optimal response to traditional endocrine therapy.

## Methods

### Cell culture

The identities of all cell lines were authenticated by DNA fingerprinting (Identifier Kit; Applied Biosystems, Grand Island, NY, USA) within 6 months of use. The BCK4 line is an ER+/AR + breast cancer line derived recently from a pleural effusion and has a nearly normal karyotype [25]. For the BCK4 cell line, the patient sample was acquired under a University of Colorado Institutional Review Board-approved tissue-acquisition protocol and patient-informed consent was obtained to acquire blood and tissue for research purposes. All other cell lines were obtained from the American Type Culture Collection; Manassas, Virginia, USA. BCK4 and MCF7 cells were grown in minimum essential media, 5% fetal bovine serum, non-essential amino acids, insulin and penicillin/streptomycin, and ZR75 cells in the same media with the addition of HEPES and l-glutamine. MCF7 cells express a wild-type AR, albeit with a shortened CAG repeat [26] that is often indicative of a more active receptor [27]. T47D cells were grown in Dulbecco’s modified Eagle’s medium supplemented with 10% fetal bovine serum, l-glutamine penicillin/streptomycin. LNCaP cells were grown in RPMI, 5% fetal bovine serum and penicillin/streptomycin. All cells were grown in a 37°C incubator with 5% carbon dioxide. MDA-MB-453 and MDA-kb2 (a derivative of MDA-MB-453 stably expressing the AR-dependent MMTV-luciferase reporter gene construct; American Type Culture Collection) were cultured in Leibovitz’s L-15 media (Invitrogen, Carlsbad, CA, USA) containing 10% fetal bovine serum (Invitrogen) and penicillin/streptomycin. MCF7-TGL cells were generated by stable infection with retroviral SFG-NES-TGL vector, encoding a triple fusion of thymidine kinase, green fluorescent protein and luciferase and sorted for green fluorescent protein.

### Tumor studies

MCF7 experiments with enzalutamide delivered in rodent chow were performed at the University of Colorado Anschutz Medical Campus and approved by the University of Colorado Institutional Animal Care and Use Committee (IACUC protocol 83611(03)1E). The MDA-MB-453 xenograft experiment in which enzalutamide was delivered by oral gavage was performed by AntiCancer Inc., San Diego, CA, USA and was approved by the Institutional Animal Care and Use Committee of AntiCancer Inc. All animal experiments were conducted in accordance with the National Institutes of Health Guidelines of Care and Use of Laboratory Animals.

For MCF7 xenograft experiments, 10^6^ MCF7-TGL cells that stably express a triple fusion of thymidine kinase, green fluorescent protein and luciferase (SFG-NES-TGL retroviral vector) for *in vivo* imaging purposes were mixed with Matrigel (BD Biosciences, Franklin Lakes, New Jersey, USA) and injected into the fourth inguinal mammary fat pad of female, ovariectomized athymic nu/nu or nonobese diabetic (NOD)/SCID mice (Taconic, Germantown, NY USA). At time of tumor injection, E2 pellets (60-day release, 1.5 mg/pellet; Innovative Research of America, Sarasota, Florida USA) or the nonaromatizable androgen 5-alpha-dihydrotestosterone (DHT) (8 mg/pellet, packed and sealed in silastic tubing) were implanted subcutaneously at the back of the neck. Tumor burden was assessed using an *in vivo* imaging system or caliper measurements (tumor volume was calculated as: length × width × depth/2). Once tumors were established, mice were matched into groups based on the total tumor burden as measured by *in vivo* imaging system or caliper. Groups receiving tamoxifen had a 90-day release, 5 mg/pellet (Innovative Research of America) implanted subcutaneously. Mice were administered enzalutamide in their chow (approximately a 50 mg/kg daily dose) or by oral gavage (10 or 25 mg/kg/day). Enzalutamide was mixed with ground mouse chow (catalog number AIN-76; Research Diets Inc., New Brunswick, NJ, USA) at 0.43 mg/g chow. The feed was irradiated and stored at 4°C before use. Mice in the control group received the same ground mouse chow but without enzalutamide. All mice were given free access to enzalutamide formulated chow or control chow during the entire study period and at an average of 3.5 g/day food intake. Feed was changed in the animal cages twice a week. Water and feed were prepared *ad libitum*. Two hours prior to sacrifice, mice were injected intraperitoneally with 50 mg/kg bromodeoxyuridine (BrdU; Sigma-Aldrich, St. Louis, MO, USA). Mice were euthanized by carbon dioxide asphyxiation followed by cervical dislocation, and the blood, tumors, colon, uteri and mammary glands were harvested.

For the MDA-453 tumor study, 6 × 10^6^ cells were injected into the fourth inguinal mammary fat pad of NOD-SCID-IL2Rgc^-/-^ female mice into which a DHT pellet (1.5 mg 60-day release; Innovative Research of America) was implanted subcutaneously. The tumor size was measured using calipers, and when tumors reached 100 mm^3^ the mice began receiving 10 mg/kg enzalutamide or vehicle by oral gavage. Once the tumors reached 400 mm^3^, another group was started on 25 mg/kg enzalutamide. At the end of the experiment, tumors were weighed and processed for embedding.

### Neoadjuvant endocrine therapy study

The inclusion criteria and trial design are described elsewhere [8,9]. Briefly, women with ER + breast cancers were enrolled in a randomized phase II clinical trial to receive exemestane alone (25 mg daily) or exemestane in combination with tamoxifen (20 mg daily) for 4 months prior to surgery. Women included in the trial were postmenopausal with newly diagnosed cancers of stage II/III, T2 to T3. Core needle biopsies were taken prior to treatment and tumor pieces from the final excision surgery were taken for analysis. The protocol was approved by the Colorado Multiple Institutional Review Board and informed consent was provided by all patients. The criteria for responders ranged from minor response to complete response, while nonresponders had stable or progressive disease.

### Tamoxifen study

This study included 192 female patients diagnosed with ER + breast cancer at the Massachusetts General Hospital (Partners) between 1977 and 1993, who were offered tamoxifen treatment as part of their adjuvant therapy and were followed at the hospital through 1998. Patients were offered tamoxifen based on estrogen positivity (≥10 pmol/mg protein) determined using either a ligand binding assay or a radioactive enzyme-linked immunosorbent assay, the standard protocol in use during this time period. As part of the present study, archival formalin-fixed paraffin-embedded tumors collected under the Institutional Review Board protocol Molecular and Cellular Predictors of Breast Cancer were stained for AR and ER by immunohistochemistry. All slides were evaluated and the percentage and intensity of both AR and ER were recorded. Each slide was also scored using the Allred scoring method.

Contingency tables were used to study the associations between the AR/ER ratio and clinicopathologic variables. In this analysis, each clinicopathologic variable was divided into two or three categories (lymph node negative vs. lymph node positive; lymph node negative vs. one to three positive vs. four or more positive; patient age <50 years vs. ≥50 years; tumor size ≤2 cm vs. >2 cm; grade 1 vs. grade 2 vs. grade 3; PR negative vs. positive; ErbB2 ≤30% vs. >30%, MIB-1 < median vs. ≥median, mitoses/10 high-powered fields (mitotic index) < median vs. ≥median, epidermal growth factor receptor  < median vs. ≥median). Patients were followed from the date of diagnosis to the date of first failure (local recurrence or distant metastasis) as well as the date of death or last follow-up. Patients who died of causes other than breast cancer and patients who were lost to follow-up or whose last encounter was before the end of the study were censored at the date of death or last encounter for survival analyses. The AR:ER ratio was calculated using a manual receiver operator characteristic analysis where we investigated the ratio that produced the best difference between good and poor prognosis in relation to disease-free survival (DFS) to identify the cutoff point for this variable. The final AR:ER ratio cutoff point was determined to be 2.0. A Fisher’s exact test was used for all dichotomized variables and the chi-square test for all trichotomized variables to compare the AR:ER ratio with other predictive markers. Kaplan–Meier curves used the calculated AR:ER ratio. All statistics were calculated using SAS (version 9.3; SAS Institute, Cary, NC, USA). Significance was determined at *P* <0.05 and all tests were two-sided.

### Immunohistochemistry

Slides were deparaffinized in a series of xylenes and ethanols, and antigens were heat retrieved in either 10 mM citrate buffer pH 6.0 (BrdU, Ki67) or 10 mM Tris/1 mM ethylenediamine tetraacetic acid buffer at pH 9.0 (AR, ER, caspase 3). Tissue for BrdU was incubated in 2 N HCl followed by 0.1 M sodium borate following antigen retrieval. Antibodies used were: AR clone 441 and ER clone 1D5 (Dakocytomation, Carpinteria, CA, USA), cleaved caspase 3 (Cell Signaling Technology, Danvers, MA, USA), Ki67 (sc-15402; Santa Cruz, Dallas, TX, USA) and BrdU (BD Biosciences, Franklin Lakes, NJ, USA). Envision horseradish peroxidase (Dakocytomation) was used for antibody detection.

Terminal deoxynucleotidyl transferase dUTP nick end-labeling (TUNEL) staining for apoptosis was performed using the ApopTag Plus Peroxidase *In Situ* Apoptosis Detection Kit (Millipore, Billerica, MA, USA), as per the manufacturer’s instructions. AR and ER staining was assessed by a pathologist (PJ or ADT) and the score is reported as intensity multiplied by percent positive cells, or in the case of the tamoxifen-treated cohort the Kaplan–Meier curve is based on percent positive cells, although results are similar and still significant when the intensity is multiplied by the percent positive cells. For ER, BrdU and TUNEL staining in xenograft studies, three separate 200× fields of each xenograft tumor were taken using an Olympus BX40 microscope (Center Valley, PA, USA) with a SPOT Insight Mosaic 4.2 camera and software (Diagnostic Instruments, Inc., Sterling Heights, MI, USA). A color threshold (RGB for positive staining nuclei, and HSB for total nuclei) was adjusted manually using ImageJ (National Institutes of Health, Bethesda, MD, USA) for each image, and particles created by the thresholds were analyzed for total area. The RGB area was divided by the HSB area and multiplied by 100 for each image. For analysis of the nuclear androgen receptor, cleaved caspase 3 and Ki67, slides were scanned at 20× on an Aperio Scan ScanScope XT, Leica Microsystems Inc. Buffalo Grove, IL United States. Mammary tumor tissue was traced separately for each tumor and necrotic areas of the tumor removed using a negative pen tool in Aperio’s Scanscope software. A nuclear algorithm was utilized to measure the percent positive cells for the Ki-67-stained and AR-stained slides and the data were exported. Cleaved caspase 3-stained slides were analyzed using a modified positive pixel count algorithm.

### Immunoblotting

Whole cell protein extracts (50 μg) were denatured, separated on SDS-PAGE gels and transferred to polyvinylidene fluoride membranes. After blocking in 3% bovine serum albumin in Tris-buffered saline–Tween, membranes were probed overnight at 4°C. Primary antibodies utilized include: ERα (Ab-16, 1:400 dilution; Neomarkers, Fremont, CA USA), AR (PG-21, 1:400 dilution; Millipore (Bedford, Massachusetts USA) or EP6704, 1:10,000; Abcam (San Francisco, CA USA), glyceraldehyde 3-phosphate dehydrogenase (1:20,000 dilution; Sigma, St. Louis, MO USA), Topo 1 (C-21, 1:100 dilution; Santa Cruz) and alpha-tubulin (clone B-5-1-2, 1:30,000 dilution; Sigma). After incubation with appropriate secondary antibody, results were detected using Western Lightning Chemiluminescence Reagent Plus (Perkin Elmer, Waltham Massachusetts USA).

### Cellular fractionation

For the MDA-kb2 cellular fractionation, cells were washed with ice-cold Dulbecco’s phosphate-buffered saline, pH 7.4, pelleted using centrifugation and resuspended in 2 volumes of ice-cold NSB (10 mM Tris · Cl, pH 7.4, 10 mM NaCl, 2 mM MgCl_2_, 1× protease inhibitors). The volume was adjusted with ice-cold NSB to 15 times the initial volume and incubated for 30 minutes on ice. The cytoplasmic fraction was obtained by addition of NP-40 to a final concentration of 0.3%. Nuclei and cytoplasm were separated using a 0.4 mm clearance Dounce homogenizer. After centrifugation, the supernatant containing the cytoplasmic fraction was collected. The pellet containing the nuclear fraction was resuspended in a 250 mM sucrose solution containing 10 mM MgCl_2_ and then 1 volume was added to 880 mM sucrose containing 5 mM MgCl_2_ under the nuclear fraction. The nuclei were then purified by centrifugation through the sucrose cushion. For the MCF7s cells, cellular fractionation was performed using the NE-PER Nuclear and Cytoplasmic Extraction Kit, Pierce Biotechnology, Rockford, IL USA as per the manufacturer’s instructions.

### Nuclear translocation assay

MDA-kb2 cells were seeded at 2 × 10^3^ cells/cm^2^ in optical microplates in Leibovitz’s L-15 medium supplemented with 5% charcoal-stripped serum. After 3 days of cultivation the cells were pretreated with enzalutamide (1 or 10 μM) for 2 hours and then co-treated with 1 nM DHT for 1 hour in the presence of enzalutamide (total 3 hours of treatment with enzalutamide). The cells were washed with phosphate-buffered saline, fixed with 4% formaldehyde for 30 minutes at room temperature and permeabilized with 0.2% triton X-100. Samples were then blocked with 5% bovine serum albumin for 1 hour and incubated with an antibody against AR (N20, sc-815 1:100; Santa Cruz) in phosphate-buffered saline 0.1% triton overnight. Incubation with the secondary antibody anti-rabbit Alexa Fluor 488 (1:1,000) was performed in 2.5% bovine serum albumin for 2 hours at ambient temperature. The nuclei were stained with 4',6-diamidino-2-phenylindole (1 μg/ml) for 30 minutes. Cells were visualized with a 60× objective and a Qimaging digital camera coupled to an Olympus X71 fluorescence microscope using a yellow fluorescent protein filter (Chroma U-N31040; Center Valley, PA, USA). The nuclear distribution of AR (ratio of nuclear AR signal/total AR signal) was quantified in a minimum of 48 cells using ImageJ software (National Institutes of Health).

### Real-time quantitative polymerase chain reaction

cDNA was synthesized from 1 μg total RNA, using M-Mulv reverse transcriptase enzyme (Promega, Fitchburg, WI, USA). For *FASN*, *PRLR* and *GCDFP-15*, SYBR green quantitative gene expression analysis was performed using the following primers: *FASN* forward, 5′-AAGGACCTGTCTGGATTTGATGC-3′ and *FASN* reverse, 5′-TGGCTTCATAGGTGACTTCCA-3′; *PRLR* forward, 5′-TATTCACTGACTTACCACAGGGA-3′ and *PRLR* reverse, 5′-CCCATCTGGTTAGTGGCATTGA-3′; *GCDFP-15* forward, 5′-TCCCAAGTCAGTACGTCCAAA-3′ and *GCDFP-15* reverse, 5′-CTGTTGGTGTAAAAGTCCCAG-3′; and 18S forward, 5′-TTGACGGAAGGGCACCACCAG-3′ and 18S reverse, 5′-GCACCACCACCCACGGAATCG-3′. For PR and stromal cell-derived factor 1 (SDF-1, also known as CXCL12), Taqman real-time polymerase chain reaction was performed using validated primer/probe sets from Applied Biosystems (assay ID: PR Hs01556702_m1, SDF-1 Hs00171022_m1, 18S Hs99999901_s1). Relative gene expression calculated using the comparative cycle threshold method and values were normalized to 18S.

### Luciferase assays

MDA-kb2 cells were plated at 5 × 10^3^ cells/well in 96-well luminescence plates and incubated overnight. Cells were treated with 10-fold serial dilutions of enzalutamide (10, 1, 0.1 μM) and DHT (10, 1, 0.1, 0.01, 0.001 nM) that were prepared in dimethylsulfoxide. Following 24 hours of incubation, the luminescence levels were determined with the luciferase assay system (Promega). Three independent experiments were performed and the luminescence values were determined as relative units and normalized to vehicle. Values are expressed as the mean fold induction ± standard error.

## Results

### A new method to examine AR relative to ER

To test the significance of AR and ER expression in breast cancer, we examined primary tumors from a group of tamoxifen-treated patients with clinical outcome data. This study included a cohort of 192 female patients diagnosed with breast cancer at the Massachusetts General Hospital (Partners) between 1977 and 1993, treated with adjuvant tamoxifen and followed at the hospital through 1998 under Institutional Review Board approval. The women ranged in age from 20 to 91 years at the time of cancer diagnosis with a median age of 68 years. Forty-eight (25.0%) of the women failed tamoxifen therapy. Women who relapsed while on tamoxifen were generally younger (median 64 years vs. 70 years for nonfailures, *P* = 0.007), had larger tumors (median 2.6 vs. 1.9 cm^3^; *P* = 0.003), had a higher proportion of grade 3 tumors (45.8% vs. 29.4%; *P* = 0.034), had more positive lymph nodes (median 2 vs. 1; *P* = 0.006), had a higher mitotic index (median 5 vs. 4; *P* = 0.007), and had lower levels of PR staining (median 5% vs. 45%, *P* = 0.048). There were no differences in MIB-1, HER2, or epidermal growth factor receptor staining percentages between the two groups. Women who failed had a median ER percent cells positive of 62.5%. This was significantly lower than the 92.5% percent cells positive in tumors that did not fail (*P* = 0.001). Although the AR percent cells positive was higher in tumors of women who failed (70% vs. 57.5% for nonfailures), the difference in AR staining percentage did not reach statistical significance.

Since we had previously observed that AR mRNA and protein decrease with treatment in tumors responsive to neoadjuvant endocrine therapy, but did not decrease in nonresponsive tumors [8,9] (Figure S1, left in Additional file 1), we decided to examine nuclear AR as compared with ER. The median AR:ER ratio in pretreatment biopsies of responsive tumors (Figure S1A in Additional file 1) in the neoadjuvant study was 1.00, with a statistically significant positive correlation between AR and ER expression (*P* = 0.006) (Figure S1A in Additional file 1). However, in nonresponsive tumors (Figure S1B in Additional file 1), the median AR:ER ratio was 3.79 with no significant correlation between AR and ER. Interestingly, in adjacent uninvolved epithelium (Figure S1C in Additional file 1), the median ratio of AR to ER expression was 0.94, again with a significant positive correlation between the two receptors (*P* = 0.0003).

Based on these intriguing results in the small neoadjuvant study, we decided to examine the amount of AR relative to ER in the larger cohort of 192 female patients diagnosed with ER + breast cancer that received adjuvant tamoxifen therapy. To identify the best cutoff point for separating patients into good and poor survival, a manual receiver operator characteristic analysis based on time to first failure (disease-free interval, DFS) was performed for the AR:ER ratio – and the optimal cutoff point of 2.0 was determined. In addition, since the AR:ER ratio was not in a log-linear relationship with the hazard function, it was necessary to use the dichotomized variable in the Cox proportional hazard models. Both AR percent cell staining and ER percent cell staining contribute to the AR:ER ratio. AR showed strong positive correlation (*r* = 0.86, *P* < 0.0001) with the ratio, while ER showed moderate negative correlation (*r* = -0.36, *P* < 0.0001). The AR:ER ratio with a cutoff value of 2.0 was significantly different between the two groups (failed tamoxifen versus nonfailed), with 27.1% of women who failed having an AR:ER ratio >2.0 compared with only 6.3% of nonfailures (*P* < 0.0001).

### High AR:ER ratio indicates poor response to traditional endocrine therapy and overall survival

We compared the correlation between AR:ER ratio (<2 or ≥2) with dichotomized study variables (Table 1). Women with the higher AR:ER ratio are more likely to have positive lymph nodes and are more likely to fail on tamoxifen. Tumors from patients with lymph node-negative disease who did not fail tamoxifen therapy (no failure within 60 months of surgery) were significantly more likely to have an AR:ER ratio less than 2.0 (*P* < 0.0001).

**Table 1:** Comparison of AR:ER ratio to clinical and pathologic variables

	**AR:ER <2**		**AR:ER ≥2**		**Chi-square**
**Variable**	***n***	**%**	***n***	**%**	***P *** **value**
Age <50	170	7.6	22	13.6	0.34
Tumor size >2 cm	170	42.9	22	59.1	0.15
Tumor grade 2 + 3	169	91.1	22	100	0.15
Lymph node-positive	133	54.1	14	85.7	**0.02**
Failed tamoxifen treatment	170	20.6	22	59.1	**<0.0001**
AR-positive	170	88.2	22	100	0.09
Progesterone receptor-positive	123	83.7	8	75.0	0.52
MIB-1 ≥21.3	168	53.0	21	23.8	**0.01**
Mitotic index number >4	165	50.3	22	45.5	0.67
erbB2 >30%	149	8.1	20	20.0	0.09
EGFR-positive	147	16.3	19	10.5	0.51

AR, androgen receptor; EGFR, epidermal growth factor receptor; ER, estrogen receptor. Bold data are significant.

**Figure 1:**
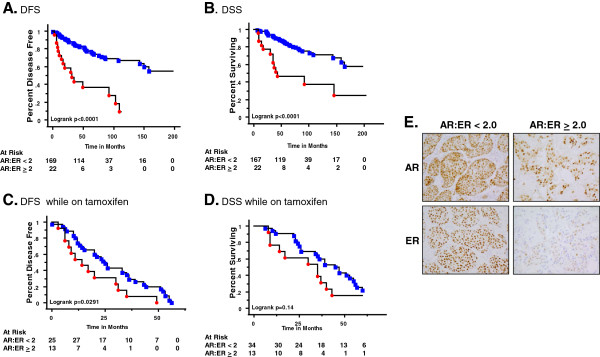
**Women with tumors having a higher AR:ER ratio have a shorter disease-free and disease-specific overall survival as compared with patients with lower AR:ER ratio.** Immunohistochemistry for androgen receptor (AR) and estrogen receptor (ER) were performed on formalin-fixed paraffin-embedded sections of primary breast cancers. Slides were scored for the percent of positive nuclear staining for AR and ER. Ratios were calculated to determine the best cutoff point for analysis. For **(A)** to **(D)** women are divided into two groups: those with AR:ER ratios <2.0 (blue squares) and those with AR:ER ratios ≥2.0 (red circles). The number of patients at risk at each time point is reflective of the number of patients censored due to no further follow-up data at each time point (underneath). Kaplan–Meier survival curve for: **(A)** disease-free survival (DFS) for all patients; **(B)** disease-specific survival (DSS) overall for all patients; **(C)** DFS for patients who failed while on tamoxifen therapy; **(D)** DSS overall for patients who failed while on tamoxifen therapy; and **(E)** representative images of AR and ER staining from the two groups (400× magnification).

We then compared study variables with tamoxifen failure by 5 years, and overall DFS and overall disease-specific survival (DSS). By univariate analyses, the tumor size, ER percent staining and AR:ER ratio were significantly associated with all survival outcomes (Table 2), while nodal positivity was significant only for tamoxifen failure and DFS. Notably, the AR:ER ratio was the most significant marker of poor survival (hazard ratio (HR) = 4.43 for tamoxifen failure, *P* < 0.0001; HR = 4.40 for DFS, *P* < 0.0001; and HR = 3.66 for DSS, *P* < 0.0001). In contrast, the ER percent cell staining was associated with reduced risk (HR = 0.98 for tamoxifen failure *P* < 0.0002; HR = 0.99 for DFS, *P* < 0.0004; and HR = 0.99 for DSS, *P* < 0.0001) (see Table 2 for 95% confidence intervals and Figure 1A,B for Kaplan–Meier curves). A number of factors were independently predictive of survival in a Cox proportional hazards model. For tamoxifen failure these variables include tumor size (HR = 1.92 for tumors >2 cm, *P* = 0.03), lymph node positivity (HR = 3.41, *P* = 0.01), and ER percent staining (HR = 0.98, *P* = 0.0002) (Table 2).

**Table 2:** Univariate analysis for associations of variables with tamoxifen failure at 5 years, disease-free survival and disease-specific survival for entire study period

	**Tamoxifen failure 5 years**			**DFS overall**		**DSS overall**	
**Variable**	***n***	**HR (95% CI)**	***P *** **value**	**HR (95% CI)**	***P *** **value**	**HR (95% CI)**	***P *** **value**
Age <50	16	1.00		1.00		1.00	
Age ≥50	175	0.49 (0.22, 1.08)	0.08	0.69 (0.33, 1.46)	0.33	0.79 (0.33, 1.87)	0.58
Tumor size ≤2 cm	105	1.00		1.00		1.00	
Tumor size >2 cm	86	1.92 (1.08, 3.42)	**0.03**	1.95 (1.18, 3.24)	**0.01**	2.39 (1.32, 4.31)	**0.004**
Tumor grade 1	15	1.00		1.00		1.00	
Tumor grade 2	112	1.78 (0.42, 7.54)	0.43	1.33 (0.47, 3.74)	0.59	1.05 (0.37, 3.02)	0.92
Tumor grade 3	63	3.33 (0.78, 14.2)	0.10	2.05 (0.71, 5.90)	0.18	1.52 (0.51, 4.51)	0.45
LN-negative	63	1.00		1.00		1.00	
LN-positive	83	3.41 (1.40, 8.31)	**0.01**	2.42 (1.19, 4.94)	**0.02**	2.12 (0.95, 4.75)	0.07
%ER, continuous	192	0.98 (0.98, 0.99)	**0.0002**	0.99 (0.98, 0.99)	**0.0004**	0.99 (0.98, 0.99)	**0.001**
AR = 0%	20	1.00		1.00		1.00	
AR >0%	171	0.61 (0.27, 1.36)	0.23	0.91 (0.42, 2.01)	0.82	1.20 (0.43, 3.33)	0.73
AR/ER < 2	169	1.00		1.00		1.00	
AR/ER ≥ 2	22	4.43 (2.33, 8.42)	**<0.0001**	4.40 (2.47, 7.83)	**<0.0001**	3.66 (1.94, 6.93)	**<0.0001**
PR-negative	22	1.00		1.00		1.00	
PR-positive	108	0.43 (0.18, 1.04)	0.06	0.62 (0.27, 1.43)	0.26	0.69 (0.26, 1.84)	0.46
MIB-1 <21.3	93	1.00		1.00		1.00	
MIB-1 ≥21.3	95	1.17 (0.66, 2.07)	0.59	0.98 (0.59, 1.61)	0.93	0.98 (0.55, 1.73)	0.94
Mitotic index number ≤4	94	1.00		1.00		1.00	
Mitotic index number >4	92	1.64 (0.92, 2.94)	0.10	1.54 (0.93, 2.55)	0.10	1.34 (0.77, 2.37)	0.30
erbB2 ≤30%	152	1.00		1.00		1.00	
erbB2 >30%	16	1.02 (0.36, 2.84)	0.98	0.71 (0.26, 1.96)	0.51	0.69 (0.22, 2.24)	0.54
EGFR = 0%	139	1.00		1.00		1.00	
EGFR >0%	26	1.31 (0.60, 2.83)	0.50	1.01 (0.50, 2.07)	0.97	1.17 (0.54, 2.51)	0.70

AR, androgen receptor; CI, confidence interval; DFS, disease-free survival; DSS, disease-specific survival; EGFR, epidermal growth factor receptor; ER, estrogen receptor; LN, lymph node; PR, progesterone receptor. Bold data are significant.

Figure 1 shows Kaplan–Meier curves with survival separated into two groups: AR:ER ratios <2.0 (blue squares) and those with ratios ≥2.0 (red circles). By the end of 10 years, the observed DFS was 10% for patients with a higher AR:ER ratio compared with approximately 70% for women with a lower ratio (Figure 1A; log-rank *P* < 0.0001). Overall, 27% (6/22) of women with high ratios remained disease free by the end of the study or at the time they were censored compared with 72% (47/169) of women with low ratios. The DSS by the end of the study was about 30% for women with higher AR:ER ratios compared with about 60% for those with lower ratios (Figure 1B; *P* < 0.0001). The majority of women with high ratios (59%; 13/22) died from their breast cancer during the study period; only 21% (36/167) of women with low ratios died (Figure 1B). As shown in Figure 1C, there is a significant difference in the time to recurrence, with patients having tumors with high AR:ER ratio failing approximately 11 months earlier than those with a low (<2) ratio. The significance does not hold up for DSS; however, patients with high AR:ER ratios died from their breast cancer on average 10 months earlier than patients with low ratios (Figure 1D). The number of patients at risk at each time point is reflective of the number of patients censored due to no further follow-up data at each time point (underneath Figure 1A,B,C,D). Representative AR/ER staining in the <2 or ≥2 categories is shown (Figure 1E).

To determine whether the AR:ER ratio was an independent predictor of poor survival, a multivariate model was used that took into account other factors known to influence outcome. Variables included in a multivariate analysis were age, grade, tumor size, ER percent staining, and the dichotomized AR:ER ratio. AR percent staining was not included in the model because it was not a significant independent predictor of failure and it was highly correlated with the AR:ER ratio (Spearman correlation coefficient, *r* = 0.86, *P* < 0.0001). Collinearity was tested for the predictor variables, particularly for ER percent staining and the AR:ER ratio. The ratio as a continuous variable was moderately negatively correlated with ER percent staining (*r* = -0.36, *P* < 0.0001) but there was no evidence of collinearity based on variance inflation analysis from linear regression models. Based on the lack of evidence for collinearity, both variables were included in the Cox models. Using a step-wise modeling strategy, the final model for tamoxifen failure consisted of the AR:ER ratio, ER percent staining and grade. Women with AR:ER ratio ≥2.0 are nearly three times more likely to fail tamoxifen therapy as compared with women with a lower ratio (HR = 2.87, *P* = 0.04; Table 3). This reflects the additional risk from the ratio above the independent effects of ER percent staining, as in this analysis the results are adjusted by the percent of ER staining and by grade. The AR:ER ratio continued to be an independent predictor of failure for DFS and DSS. The hazard ratio for the dichotomized AR:ER ratio was higher for DFS (HR = 4.04, *P* = 0.002). For DSS, the measure of effect was slightly lower (HR = 2.75, *P* = 0.03). Both DFS and DSS models were adjusted for ER percent staining and tumor size.

**Table 3:** Multivariate Cox proportional hazards models for tamoxifen failure at 5 years, disease-free survival and disease-specific survival for entire study period

	**AR:ER ratio ≥ 2**					
	***n***	**Events**	**HR**	**95% CI**	***P *** **value**	**Model adjusted by**
Tamoxifen failure at 5 years	191*	48	2.87	1.08, 7.67	**0.04**	ER%, tumor grade
DFS overall	191**	63	4.04	1.68, 9.69	**0.002**	ER%, tumor size
DSS overall	190**	49	2.75	1.11, 6.86	**0.03**	ER%, tumor size

AR, androgen receptor; CI, confidence interval; DFS, disease-free survival; DSS, disease-specific survival; ER, estrogen receptor. Bold data are significant.

*One case was missing tumor grade.

**Missing outcome data.

To investigate whether the AR:ER ratio was merely a reflection of the level of ER positivity, we tested various cutoff points for ER% cell staining. Using 20% cell staining positive for the ER cutoff point, we determined that although those patients with little ER were of course more likely to have a high AR:ER ratio (10/15), there were 12/165 tumors with high ER levels that also had a high AR:ER ratio (>2.0). A high AR:ER ratio is therefore not merely a consequence of low ER. In the multivariate setting, while the dichotomization of ER at <20% versus ≥20% was significant alone, when the AR:ER ratio was added ER percent cell staining lost its significance.

### Androgens are proliferative in ER+/AR + breast cancer lines and the AR signaling inhibitor enzalutamide inhibits androgen-mediated proliferation and tumor growth *in vivo*

**Figure 2:**
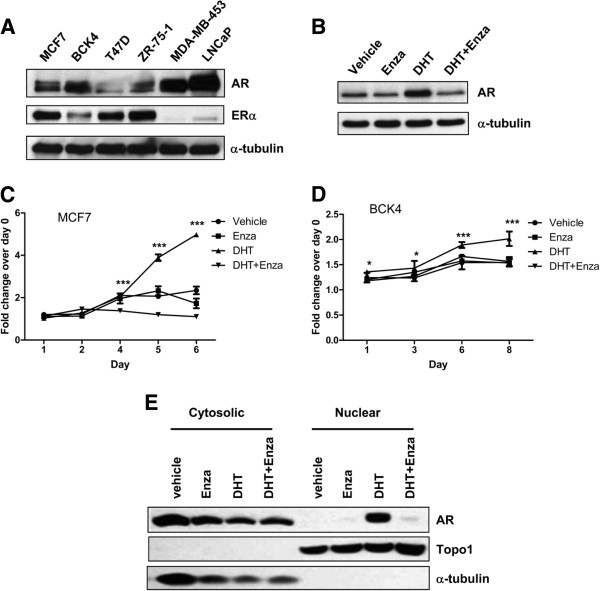
**Enzalutamide abrogates androgen mediated proliferation in estrogen receptor-positive breast cancer cells. (A)** Baseline levels of androgen receptor (AR) and estrogen receptor (ER) alpha protein in whole cell lysates from ER-positive (MCF7, BCK4, T47D and ZR-75-1) and ER-negative (MDA-MB-453) breast cancer and prostate (LNCaP) cancer cell lines. **(B)** AR protein levels in MCF7 cells plated in charcoal-stripped serum-containing media for 48 hours prior to treatment with vehicle control, 10 nM 5-alpha-dihydrotestosterone (DHT), 10 μM enzalutamide (Enza) or a combination of DHT and Enza for 48 hours. **(C)** MCF7 and **(D)** BCK4 breast cancer cells, both ER + AR+, were treated with vehicle control, 10 nM DHT, 10 μM Enza or a combination of DHT and Enza, and MTS proliferation assays were performed. Error bars represent standard error of the mean. *P < 0.05, ***P < 0.001 for DHT versus DHT + Enza, analysis of variance with Bonferroni’s multiple comparison test correction. **(E)** AR levels in cytosolic and nuclear fractions of MCF7 cells treated with vehicle, 10 nM DHT, 10 μM enzalutamide or DHT + Enza for 3 hours.

Lysates from four luminal ER + breast cancer cell lines were probed for AR and ER (Figure 2A). The prostate cancer cell line LNCaP and the molecular apocrine breast cancer cell line MDA-MB-453, which express high levels of AR [20,28,29], were used as positive controls for AR expression. MCF7 cells and the newly derived BCK4 cell line express both AR and ER (Figure 2A) and the new androgen receptor signaling inhibitor enzalutamide prevents ligand-mediated stabilization of AR protein in MCF7 cells (Figure 2B). Both cell lines proliferate in response to DHT (Figure 2C,D). Unlike androstenedione and testosterone, DHT is not aromatizable to estrone or E2 [30-32]. DHT-stimulated proliferation was blocked by enzalutamide in both the MCF7 and BCK4 lines (Figure 2C,D). Enzalutamide inhibited DHT-mediated nuclear translocation of AR within 3 hours as determined by nuclear and cytosolic fractionation (Figure 2E).

**Figure 3:**
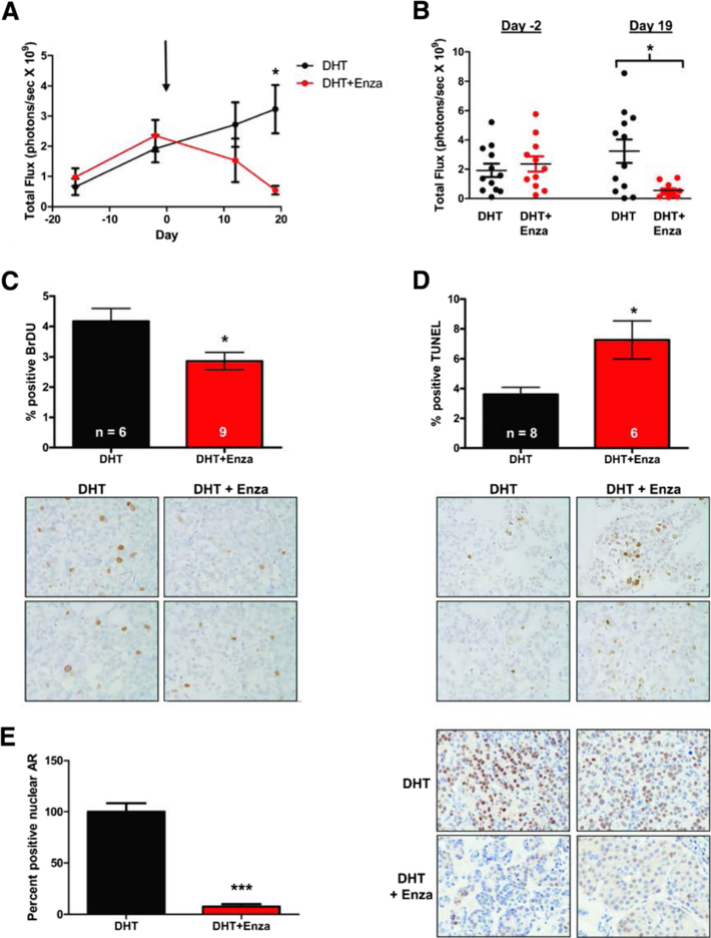
**Enzalutamide inhibits androgen-stimulated growth of MCF7 tumors *in vivo*.** MCF7-TGL cells stably expressing luciferase were implanted orthotopically in the mammary gland of NOD/SCID ovariectomized female mice with a 5-alpha-dihydrotestosterone (DHT) pellet implanted subcutaneously. Mice were matched into two groups based on tumor volume (day -2) and treatment with either control chow (DHT) or chow containing 50 mg/kg enzalutamide (DHT + Enza) begun (day 0, indicated by arrow), and the tumor burden was measured by whole-body luminescence. **(A)** Mean total flux of all mice in each of the treatment groups. Error bar represents standard error of the mean. *P < 0.05, Wilcoxon rank sum. **(B)** Total luminescent flux is shown for all individual mice at the day of matching (day -2) and at the final imaging day (day 19). *P < 0.05, Wilcoxon rank sum. **(C)** Mice were injected with bromodeoxyuridine (BrdU) 2 hours prior to sacrifice and BrdU immunohistochemistry was performed on formalin-fixed paraffin-embedded tumor sections and quantified. *P < 0.05, Student’s *t* test. Representative images of BrdU staining (400× magnification) and quantification. **(D)** Quantification of apoptotic cells as measured by terminal deoxynucleotidyl transferase dUTP nick end-labeling (TUNEL) staining with representative images below (400× magnification). *P < 0.05, Student’s *t* test. **(E)** Quantification of nuclear AR staining and representative images (400× magnification). ***P < 0.001, Wilcoxon rank sum.

To determine whether enzalutamide inhibits androgen-mediated growth *in vivo*, MCF7 cells constitutively expressing luciferase (MCF7-TGL) were injected into the mammary fat pad of ovariectomized mice implanted with DHT pellets and the tumor burden was measured using luminescent imaging and caliper measurements. Once tumors were established, mice were matched based upon tumor imaging (day -2) into two treatment groups, one receiving control chow and the other receiving chow containing 50 mg/kg enzalutamide on day 0. Tumors in the DHT-treated mice on control chow continued to grow, while mice receiving DHT plus enzalutamide showed regression of tumors by the *in vivo* imaging system (Figure 3A) and caliper measurement (data not shown). On the final day of imaging (day 19), tumors had regressed to near undetectable levels, with an 83.2% decrease in luminescence in mice receiving DHT plus enzalutamide as compared with the DHT group (Figure 3A,B). As determined by BrdU incorporation and immunostaining, proliferation in the enzalutamide-treated tumors was 31.3% lower than in tumors treated with DHT alone (Figure 3C). TUNEL staining indicated a 50% increase in apoptotic cells in enzalutamide-treated tumors (Figure 3D). A dramatic (92.5%) decrease in AR nuclear localization was observed in the tumors treated with enzalutamide (Figure 3E), consistent with the ability of enzalutamide to impair nuclear entry of AR in prostate cancer [33]. Similar results to the above with MCF7 xenografts were obtained in mice administered enzalutamide by oral gavage, where tumor burden decreased in a dose-dependent manner (data not shown).

### Enzalutamide inhibits androgen-mediated growth in ER– breast cancer cells *in vitro* and *in vivo*

The MDA-MB-453 breast cancer cell line represents the ER– molecular apocrine or luminal androgen receptor subtype of breast cancer with high levels of AR [17,18,20,34]. In this line, AR contains a point mutation (Q865H) reported to decrease sensitivity to DHT [35]. Nonetheless, these cells proliferate in response to androgens [28,29] and we therefore sought to determine whether enzalutamide could block DHT-mediated effects on proliferation and gene expression. Indeed, enzalutamide completely abrogated proliferation induced by DHT (Figure S2A in Additional file 2) and expression of known AR-regulated genes [29], such as fatty acid synthase, gross cystic disease fluid protein (also called prolactin inducible protein) and prolactin receptor, was reduced by enzalutamide (Figure S2B in Additional file 2). Further, in MDA-MB-453 cells that stably express an androgen responsive luciferase reporter (MDA-kb2) [36], enzalutamide inhibited luciferase reporter activity in a dose-dependent manner (Figure S2C in Additional file 2). Enzalutamide impairs ligand-mediated nuclear import of AR in prostate cells [33], and in MDA-kb2 cells it reduced the ratio of nuclear to total AR (Figure S2D in Additional file 2). Immunoblotting for AR in nuclear and cytoplasmic lysates demonstrates that the same is true in wild-type MDA-MB-453 cells (Figure S3 in Additional file 3).

**Figure 4:**
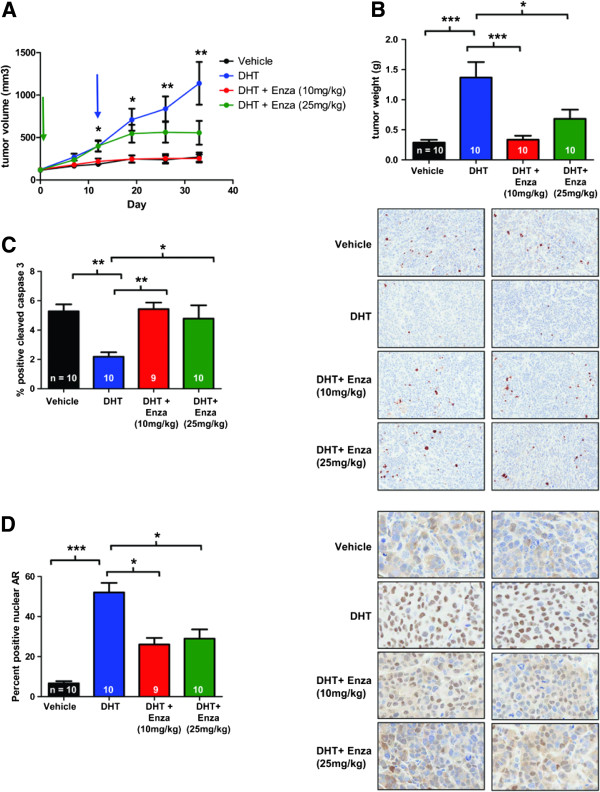
**Enzalutamide inhibits androgen-stimulated growth of MDA-MB-453 tumors.** MDA-MB-453 cells were injected orthotopically in the mammary gland of female NOD-SCID-IL2Rgc^-/-^ mice. Three groups had a 5-alpha-dihydrotestosterone (DHT) pellet implanted subcutaneously and one group had no pellet (Vehicle). Once tumors reached an average size of 100 mm^3^ (green arrow), mice were given either enzalutamide (Enza, 10 mg/kg) or vehicle (Vehicle and DHT groups) by daily oral gavage. Another group was given a higher dose of Enza (25 mg/kg) by oral gavage when tumors reached an average size of 400 mm^3^ (blue arrow). **(A)** Tumor volume was measured weekly by caliper. Error bars represent standard error of the mean. *P < 0.05, **P < 0.01 for DHT versus DHT + Enza (10 mg/kg), Wilcoxon rank sum. **(B)** Tumors were excised and weighed at the end of the experiment. **(C)** Tumor sections stained for cleaved caspase 3 were quantified (left) and representative images shown (right) (200× magnification). *P < 0.05, **P < 0.01, ***P < 0.001, analysis of variance with Bonferroni’s multiple comparison test correction. **(D)** Nuclear androgen receptor staining was quantified (left) and representative images (400× magnification) are shown (right). *P < 0.05, ***P < 0.001, Kruskal–Wallis with Dunn’s multiple comparison test correction.

To determine whether enzalutamide inhibits androgen-induced tumor growth of ER– breast cancer cells, MDA-MB-453 xenografts were grown at the orthotopic site in mice implanted with a DHT pellet and the tumor size was measured by caliper. Once tumors reached 100 mm^3^, mice were treated with 10 mg/kg/day enzalutamide or vehicle by oral gavage (Figure 4A, green arrow). DHT stimulates tumor growth as previously reported [20], but in mice treated with DHT plus enzalutamide (10 mg/kg by oral gavage) tumors did not significantly differ from mice that received vehicle control (Figure 4A). Another group of mice received a higher dose of enzalutamide (25 mg/kg/day) starting when tumors reached an average of 400 mm^3^ (Figure 4A, blue arrow). At this higher dose, there was a trend towards decreased tumor size, although this did not reach statistical significance (Figure 4A). Tumor weights in either the low-dose or high-dose enzalutamide treatments were significantly lower than mice treated with DHT only, an 85.2% and 65.0% decrease respectively (Figure 4B), indicating that the caliper measurements for a high dose of enzalutamide underestimates the decreased tumor burden in this group. Interestingly, there was a statistically significant increase in apoptosis in both enzalutamide treatment groups versus DHT (60.0% and 54.3% increase in low-dose and high-dose groups respectively), as measured by cleaved caspase 3 (Figure 4C, quantification on left and representative images on right), but there was no difference in the proliferation rate of any of the groups, as measured by Ki67 staining (not shown). Thus, in MDA-MB-453 tumors, DHT protects cells against apoptosis and enzalutamide impairs this anti-apoptotic effect. Consistent with the *in vitro* data, enzalutamide decreased ligand-mediated nuclear entry of AR such that there is a significant decrease (50.0% in low dose and 44.3% in high dose) in the number of AR-positive nuclei in the enzalutamide-treated tumors (Figure 4D, quantification on left and representative images on right). Similarly, when an MDA-MB-453 xenograft study was performed with low-dose and high-dose enzalutamide treatments initiated when the tumors reached 100 mm^3^ (Figure S4A in Additional file 4), tumor growth was decreased in a dose-dependent manner (Figure S4B in Additional file 4) and was associated with significantly reduced nuclear AR staining in enzalutamide-treated tumors (Figure S4C in Additional file 4). Steady-state concentrations of enzalutamide, including the pharmacologically active metabolite *N*-desmethyl-MDV3100, in the MDA-MB-453 xenograft studies were only moderately lower than what has been reported in patients receiving 160 mg/day enzalutamide (Cmax values for enzalutamide and the pharmacologically active metabolite, *N*-desmethyl enzalutamide, were 16.6 μg/ml and 12.7 μg/ml, respectively).

### Enzalutamide inhibits estrogen mediated growth *in vitro* and *in vivo*

**Figure 5:**
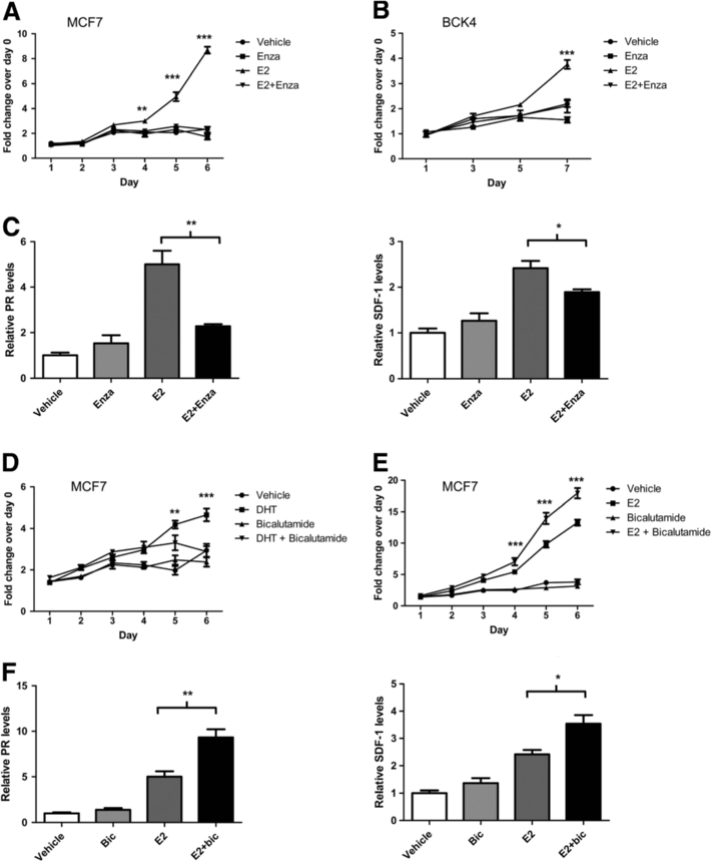
**Enzalutamide inhibits estradiol-mediated proliferation of breast cancer cells, while bicalutamide does not.** MTS proliferation assays were performed on **(A)** MCF7s cells and **(B)** BCK4 cells treated with vehicle control, 10 nM estradiol (E2), 10 μM enzalutamide (Enza) or a combination of E2 and Enza. Error bars represent standard error of the mean (SEM). **P < 0.01, ***P < 0.001 for E2 versus E2 + Enza, analysis of variance (ANOVA) with Bonferroni’s multiple comparison test correction. **(C)** MCF7 cells were treated for 48 hours with treatments as above and real-time polymerase chain reaction (PCR) was performed for estrogen-responsive genes, progesterone receptor (PR) and stromal cell-derived factor 1 (SDF-1, also known as CXCL12). Each gene is normalized to 18S and shown relative to vehicle. *P < 0.05, ***P < 0.001, Student’s *t* test. MCF7 cells were treated with vehicle control, **(D)** 1 μM bicalutamide, 10 nM 5-alpha-dihydrotestosterone (DHT) and DHT + bicalutamide or **(E)** with 10 nM E2 and E2 + bicalutamide. **P < 0.01, ***P < 0.001 for DHT versus DHT + bicalutamide, or E2 versus E2 + bicalutamide, ANOVA with Bonferroni’s multiple comparison test correction. **(F)** MCF7 cells treated for 48 hours with vehicle, 1 μM bicalutamide, 10 nM E2 and E2 + bicalutamide and real-time PCR performed for PR and SDF-1. *P < 0.05, ***P < 0.001, Student’s *t* test. Error bars represent SEM, Student’s *t* test (all analyses).

While enzalutamide has high affinity binding for AR, it does not significantly bind to either ERα or ERβ as determined by ligand binding assays (Table S1 in Additional file 5). However, originally as a negative control in experiments where we were antagonizing DHT with enzalutamide, we combined enzalutamide with E2 in ER+/AR + breast cancer cells. Surprisingly, enzalutamide significantly inhibited E2-induced proliferation of both MCF7 and BCK4 cells *in vitro* (Figure 5A,B). Enzalutamide also inhibited E2-induced upregulation of PR and SDF-1, two estrogen-responsive genes (Figure 5C). In stark contrast, although bicalutamide effectively inhibited DHT-mediated proliferation in MCF7 cells (Figure 5D), it had the opposite effect on E2 signaling, as it significantly increased E2-mediated proliferation (Figure 5E) and increased the E2-mediated induction of PR and SDF-1 mRNA (Figure 5F).

**Figure 6:**
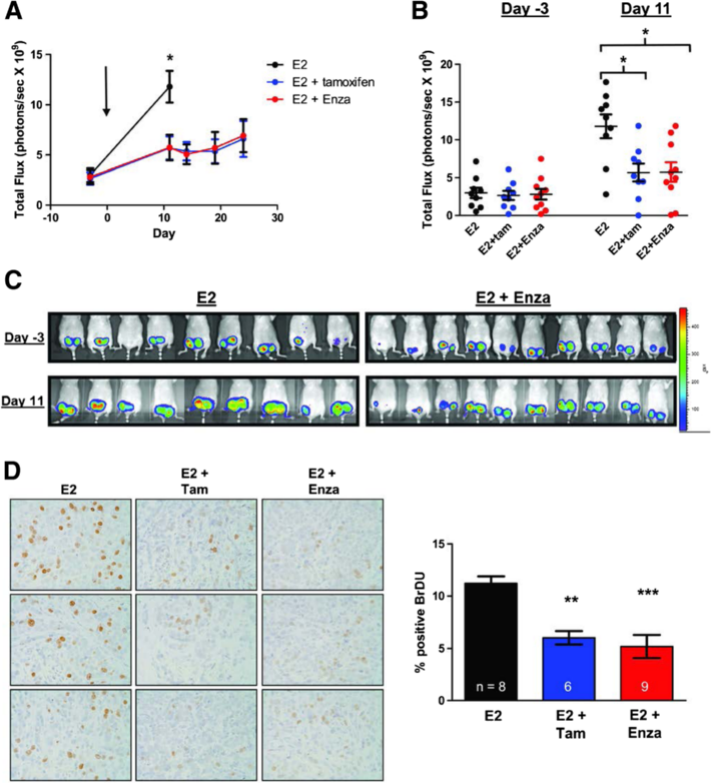
**Enzalutamide inhibits estrogen-stimulated growth of MCF7 tumors as effectively as tamoxifen.** MCF7-TGL cells stably expressing luciferase were implanted orthotopically in the mammary gland of ovariectomized female nude mice. All mice had an estradiol (E2) pellet implanted subcutaneously and were given either control chow (E2), control chow plus a tamoxifen pellet implanted subcutaneously (E2 + Tam) or chow containing 50 mg/kg enzalutamide (E2 + Enza). The tumor burden was measured by whole-body luminescence. **(A)** Mean total flux. Mice were matched on day -3 and treatment began on day 0 (arrow). *P < 0.05, analysis of variance (ANOVA) with Bonferroni’s multiple comparison test correction. **(B)** The total luminescent flux is shown for individual mice on the day of matching (day -3) and of final imaging (day 11). *P < 0.05, ANOVA with Bonferroni’s multiple comparison test correction. **(C)** Images of luminescent signal in the two treatment groups at time of matching (day -3) and the final day of imaging (day 11). **(D)** Mice were injected with bromodeoxyuridine (BrdU) 2 hours prior to sacrifice and immunohistochemistry for BrdU was performed on tumor sections and quantified using imageJ (National Institutes of Health, Bethesda, MD, USA). Representative images of BrdU staining (left, 400× magnification) and quantification (right). **P < 0.01 for E2 versus E2 + Tam, ***P < 0.001 for E2 versus E2 + Enza, ANOVA with Bonferroni’s multiple comparison test correction.

To determine the effect of enzalutamide on E2-stimulated breast tumor growth *in vivo*, a xenograft study was performed injecting MCF7-TGL cells in ovariectomized mice implanted with an E2 pellet. Cells were injected orthotopically and once tumors were established (arrow, average size of 100 mm^3^), mice were matched into three groups: control chow; control chow and a tamoxifen pellet; and chow containing 50 mg/kg enzalutamide (Figure 6A). Enzalutamide significantly inhibited E2-mediated MCF7 tumor growth as effectively as tamoxifen, with a decrease in tumor luminescence of 59.9% for the tamoxifen group and 70.3% in the enzalutamide group at day 11. Day 11 was the final day of imaging for the E2-only group since these mice had to be euthanized due to tumor burden. Luminescence flux for individual animals (Figure 6B) and images of mice (Figure 6C) are shown for the day of matching (day -3) and the last imaging day when all mice were alive (day 11). Both drugs significantly decreased cell proliferation, with a 46.4% decrease in the E2 plus tamoxifen group and a 54.2% decrease in the E2 plus enzalutamide group compared with the E2 group, as measured by BrdU incorporation (Figure 6D). In contrast to what was observed in DHT-mediated tumor growth, enzalutamide did not increase apoptosis when opposing E2-stimulated growth (data not shown). Interestingly, ER protein levels in the MCF7 xenograft tumors were affected differently by tamoxifen versus enzalutamide (Figure S5 in Additional file 6). ER immunostaining was quantified with ImageJ and by pathologist (PJ) scoring in a blinded manner for percent cells positive for nuclear ER. By both methods, ER was extremely low in the E2-alone group, but significantly increased with the addition of tamoxifen. However, in the E2 plus enzalutamide group, ER levels are not significantly different from E2 alone, indicating that enzalutamide does not elicit upregulation of ER like tamoxifen (Figure S5 in Additional file 6) and suggests that enzalutamide affects ER by a different mechanism than the competitive antagonist tamoxifen. This intriguing finding will be the focus of a subsequent study.

Importantly, mean animal weights during and at the end of all *in vivo* studies showed no differences across treatment groups, indicating no adverse effects on the general health of the mice (Figure S6 in Additional file 7).

## Discussion

The vast majority of ER + breast cancers are clearly also AR + (84 to 91%) [5,37,38] and patients with tumors that co-express AR with ER and PR have a longer DFS than those with tumors negative for all three receptors [37], probably reflecting a more well-differentiated state than that of receptor-negative tumors . However, the question of whether androgens and ARs are harmful or beneficial for patients with breast cancer is complex [39-41] and probably differs with menopausal status, treatment and breast cancer subtype.

Our analysis of 192 women with ER + breast cancers treated with tamoxifen revealed that rather than the level of AR expression, the AR:ER ratio may play a role in disease progression and response to treatment. In our cohort, women with tumors expressing a high ratio of AR:ER (≥2.0) had over four times the risk for failure while on tamoxifen (HR = 4.43) compared with women with a low ratio (<2.0). When ER percent cell staining was added to the model, the risk dropped to 2.87-fold, showing that although ER percent staining explained some of the increase in risk from a higher ratio, the AR:ER ratio actually has an independent effect on risk for failure above ER percent staining. In summary, the data indicate that a high ratio of nuclear AR to ER protein is indicative of shorter time to relapse in patients treated with tamoxifen, and may also be indicative of a lack of response to neoadjuvant AI treatment. Although they need to be tested in additional cohorts, these provocative findings suggest that the AR:ER ratio may be a new, independent predictor of response to traditional E2/ER-directed endocrine therapies. The finding may also indicate that patients that relapse while on tamoxifen or AIs might be good candidates for AR-directed therapy. Lastly, AR:ER ratio is also an independent predictor of DFS (HR = 4.04, 95% confidence interval: 1.68, 9.69; *P* = 0.002) and DSS (HR = 2.75, 95% confidence interval: 1.11, 6.86; *P* = 0.03).

Our *in vitro* and preclinical results demonstrate that enzalutamide inhibits androgen-stimulated growth of both ER+/AR + and ER–/AR + breast tumors. Surprisingly, with regard to E2-mediated proliferation, enzalutamide, which works by impairing androgen-mediated AR nuclear entry, gives a completely different result than the traditional anti-androgen, bicalutamide. Although DHT is clearly proliferative in MCF7 and BCK4 cells, in some breast cancer cell lines DHT decreased E2-induced proliferation [28,42-44]; however, the antagonist bicalutamide consistently increased E2-mediated proliferation. This bicalutamide-mediated increase in E2-stimulated proliferation was interpreted as indicating that AR is protective against E2-mediated breast cancer cell proliferation. However, we now present contrasting results demonstrating that inhibition of AR with enzalutamide decreases ER-mediated proliferation. A critical difference between the two drugs is that while bicalutamide permits AR nuclear entry, enzalutamide greatly impairs AR localization and ligand-mediated stabilization, as indicated in studies in prostate cancer and our nuclear and cytosolic fractionation and immunohistochemistry in xenograft tumors presented in this study. Our results with enzalutamide thus shed new light on the role of AR in breast cancer, since *in vitro* and *in vivo* preclinical studies demonstrate that inhibiting AR nuclear localization decreases both androgen and estrogen-stimulated tumor growth.

We propose an explanation that reconciles conflicting reports regarding the role of AR in breast cancer by recognizing that hormonal influences on the breast are quite different in premenopausal versus postmenopausal women. Data suggesting a protective effect of androgens studied androgen in the presence of estrogen, thereby more closely modeling the premenopausal state [45] where androgens and AR may be protective against E2-mediated proliferation. AR can bind to the ER cofactor FOXA1 and to estrogen response elements, albeit as a weaker transcriptional activator than ER at these loci; therefore, the net effect of liganded AR competing with liganded ER may be decreased E2-mediated proliferation [42]. Additionally, in ER–/AR + tumors such as the MDA-MB-453 cell line, global AR binding events largely overlap that of ER in ER + luminal A tumors [19]. In contrast, in postmenopausal women with ER + breast cancer (which represent the majority of cases), and particularly in those being treated with AIs, circulating levels of E2 are extremely low, while circulating androgen levels are slightly elevated since AIs block the conversion of androgens to estrogen [12]. Importantly, circulating levels of testosterone, androstenedione, and DHEA-S increase in women on AI therapy [13] as compared with pretreatment levels. Furthermore, high levels of the adrenal androgen DHEA-S before treatment are predictive of failure on AIs and circulating DHEA-S increased during treatment in patients with tumors that failed to respond to AI treatment [14]. In the context of a postmenopausal woman on AI therapy (in the absence of estrogen), it is possible that activated AR could mediate protumorigenic pathways in breast cancers. As recently reviewed [40,46], the data in cell lines regarding whether DHT is proliferative are very conflicting; however, a study with seven lines derived from ductal carcinomas demonstrated that the majority were growth stimulated by physiologic levels of testosterone [47]. Interestingly, local production of sex steroids can occur, and DHT levels have been found to be significantly higher in carcinomatous breast tissues than in the blood of postmenopausal breast cancer patients [48].

DHT is not aromatizable [31,32,49], indicating that conversion to estrogens is not causing breast tumor growth in our study. Furthermore, we observe that enzalutamide acts differently when it opposes DHT versus E2-driven tumor growth. Enzalutamide very effectively blocks DHT-mediated protection against apoptosis in both ER + and ER– tumors, but it inhibits proliferation but does not affect apoptosis when opposing E2-stimulated tumor growth in ER+/AR + models. Although enzalutamide does not bind ER, it appears to affect ER in MCF7 xenograft tumors, but in a different manner than tamoxifen. Furthermore, we find that enzalutamide blocks the E2-mediated induction of ER-regulated genes such as the chemokine SDF-1 (also known as CXCL12). SDF-1 mediates the mitogenic effects of E2 in breast cancer cells [50]. The SDF-1/CXCR4 pathway can activate ER via phosphorylation, and E2-driven proliferation is blocked by inhibition of this pathway [51]. SDF-1 promotes the growth of prostate epithelial cells by promoting the nuclear localization of AR, binding of AR to DNA and increased PSA protein in a ligand independent manner [52]. In contrast to enzalutamide, bicalutamide enhances upregulation of SDF-1 and other E2-regulated genes, and enhances E2-mediated breast cancer cell proliferation. This difference in how enzalutamide and bicalutamide affect ER activity may provide insight into the role of AR in breast cancer. When bound to bicalutamide, AR can still translocate to the nucleus and bind to DNA [22]. In contrast, enzalutamide has been reported to impair liganded AR nuclear entry in prostate cancer cells [33,53], as we see in this study in breast cancer cell lines in culture and xenografts. Our observation that enzalutamide blocks E2-induced proliferation and inhibits liganded ER activity on classical ER-regulated genes thus suggests that nuclear AR is critical for ER function. Indeed, AR and ER can directly interact in breast cancer cells [54,55].

## Conclusion

While AR has been considered a potential therapeutic target in ER–/AR + breast cancers [2,17,18,20], it has not previously been suggested as a target in ER + breast cancers. Our data in clinical specimens suggest that the ratio of nuclear AR to ER may critically influence tumor biology and response to endocrine therapy. A high AR:ER ratio may be predictive of suboptimal response to ER-directed endocrine therapy. Furthermore, higher nuclear expression of AR relative to ER may also be indicative of active AR, since AR translocates to the nucleus and is stabilized upon ligand binding. AR and ER are expressed at roughly equivalent amounts in tumors that respond to neoadjuvant endocrine therapy and in adjacent uninvolved epithelium, suggesting that similar levels of AR and ER reflect a more normal state. In addition to being a predictor of poorer response to traditional endocrine therapy and overall DFS, high levels of AR relative to ER may also identify a subset of breast cancers that would respond more favorably to enzalutamide alone or combined with tamoxifen or AIs. Targeting AR may prove useful in patients with recurrent ER + disease. If the long-term selective pressure of drugs targeting the E2/ER pathway leads to tumors switching to dependence on androgens, initial treatment with both AI and enzalutamide may be beneficial. In summary, our preclinical data support the initiation of clinical studies evaluating enzalutamide for treatment of AR + tumors regardless of ER status, since enzalutamide uniquely blocks both androgen-mediated and estrogen-mediated tumor growth. Recently, a mutation was discovered in AR that confers resistance to enzalutamide and another new generation anti-androgen, ARN-509, [56,57]. Whether such mutations will also arise in breast cancer patients treated with anti-androgens remains to be seen.

## Abbreviations

AI: aromatase inhibitor; AR: androgen receptor; BrdU: bromodeoxyuridine; DFS: disease-free survival; DHEA-S: dehydroepiandrosterone sulfate; DHT: 5-alpha-dihydrotestosterone; DSS: disease-specific survival; E2: estradiol; ER: estrogen receptor; HR: hazard ratio; NOD-SCID: nonobese diabetic, severe combined immunodeficiency; PR: progesterone receptor; SDF-1: stromal cell-derived factor 1; TUNEL: terminal deoxynucleotidyl transferase dUTP nick end-labeling.

## Competing interests

AAP, SB, and SM are full-time employees of Medivation. JG, FG, IEA, and EMcC are employed by Fundacion Ciencia & Vida in Santiago, Chile and receive partial funding from Medivation. The remaining authors declare that they have no competing interests. Enzalutamide is being co-developed by Medivation, Inc. and Astellas.

## Authors’ contributions

DRC performed proliferation assays, reverse transcriptase-polymerase chain reaction, tumor imaging, necropsies, statistical analysis of xenograft experiments and drafted the manuscript. SB directed the work at FCV in Chile, and contributed to study conception and design. BMJ performed BCK4 proliferation assays, assisted with xenograft surgeries, experimental design of in *vitro* and *in vivo* assays at Universit of Colorado (UC) and revised the manuscript. DMC conducted imaging and assisted with statistical analysis of xenograft experiments at UC. ENH assisted with xenograft studies (imaging and necropsies) at UC. NCD performed westerns and nuclear and cytosolic fractionations. NSS performed all tissue processing for preclinical histology, immunohistochemistry, TUNEL assays, and ImageJ analysis for xenograft studies at UC. SME identified tamoxifen treatment patient clinical specimens, collected clinical follow-up data, calculated AR:ER percent cell positive ratios, and performed statistics for the archival tamoxifen AR study. AJ performed proliferation assays, western assays, cellular fractionation, imaging and measurements of xenograft assays. JG carried out *in vitro* experiments including cell proliferation, cell imaging and fractionation. FG conducted molecular biology experiments and quantitative reverse transcriptase-polymerase chain reaction of MDA-MB-453 experiments. SM assisted in the design of dose selection and interpretation of *in vivo* data. IEA and EMcC contributed to the design of *in vivo* MDA-MB-231 xenografts, analysis and interpretation of *in vitro* and *in vivo* data performed in Chile. Pathologist PJ performed pathological examination of preclinical tissue samples. KCT performed or reviewed all statistical analysis of clinical data. ADT, breast pathologist, was principal investigator for the archival tamoxifen dataset used and retains overall responsibility for the database, reviewed immunohistochemistry for this study and all of the original tissue sections for diagnosis and tumor characteristics from over 1,200 patients to identify tissue sections usable for research. Oncologist ADE was the principal investigator of the AI clinical trial and also made substantial intellectual contributions to conception and design of preclinical studies. AAP contributed to the design of tissue culture and preclinical studies and assisted in the interpretation of data. JKR was principal investigator of laboratory studies conducted at UC, was responsible for overall conception and design of studies, collection, review, and interpretation of data and writing of the manuscript. All authors read and revised the manuscript critically for intellectual content and approved the final manuscript. 

## Supplementary Material

Additional file 1: Figure S1 Showing breast tumors that respond to endocrine therapy tend to have decreased AR expression while nonresponders tend to maintain AR expression. There is a positive correlation between AR and ER in responsive tumors and uninvolved adjacent epithelium. Patients received 4 months of neoadjuvant endocrine therapy (exemestane or exemestane + tamoxifen). Core biopsies taken prior to treatment (pre) and a tumor sample at the time of surgery (post) were stained for AR expression. Graph depicts the AR score (percent cells positive for nuclear AR staining versus intensity) in the pre and post treatment samples for those who responded to the endocrine therapy versus nonresponders. *P* = 0.064, Wilcoxon matched-pair test (left top). Staining of AR in representative responsive and nonresponsive tumors pre versus post treatment is shown below (400× magnification) (left, bottom). In the same tumors, staining score (percent positive staining × intensity) for nuclear AR was plotted on the *y* axis and ER on the *x* axis for patients who responded (A, graph) versus those who did not (B, graph). Normal uninvolved glands adjacent to tumors were scored for AR and ER (C, graph). The slope of the line (β) is indicated, as well as the *P* value, Spearman correlation. Representative images of AR and ER staining (400× magnification) in responders (A, right), nonresponders (B, right) and normal adjacent (C, right) (1,000× magnification). Click here for file

Additional file 2: Figure S2 Showing that enzalutamide (Enza) abrogates DHT-mediated proliferation in ER-negative breast cancer cells. (A) MTS proliferation assays were performed in MDA-MB-453 cells treated with vehicle, 10 nM DHT, 10 μM Enza or DHT + Enza. Error bars = standard error of the mean (SEM). (B) Real-time polymerase chain reaction for androgen responsive genes fatty acid synthase (FASN), gross cystic disease fluid protein (GCDFP-15, also called prolactin inducible protein) and prolactin receptor (PRLR) was performed from RNA harvested from MDA-MB-453 breast cancer cells treated with vehicle, 10 μM Enza, 10 nM DHT or DHT + Enza for 24 hours. Genes normalized to 18S and relative to vehicle. *P < 0.05, **P < 0.01 for Student’s *t* test. (C) MDA-k2b cells, which contain an androgen responsive luciferase construct, were treated for 24 hours with various concentrations of DHT alone or in combination with 1 or 10 μM Enza prior to luciferase assay, and luciferase units relative to the 0.001 nM DHT are shown. Error bars = SEM. (D) MDA-kb2 cells were treated as indicated for 3 hours. Nuclear and total AR staining was quantified with graph indicating the ratio of nuclear to total AR (each triangle represents one cell). Representative images (600× magnification). For proliferation and luciferase assays and the quantification of nuclear/total AR ratio, *P < 0.05, **P < 0.01, ***P < 0.001 for DHT versus DHT + Enza, analysis of variance with Bonferroni’s multiple comparison test correction. Click here for file

Additional file 3: Figure S3 Showing that enzalutamide (Enza) impairs DHT-mediated nuclear entry of AR in apocrine breast cancer cells. MDA-453 cells were treated with vehicle, 10 nM DHT, 10 μM enzalutamide or DHT + Enza for 3 hours. After nuclear and cytoplasmic fractionation, lysates were immunoblotted for AR, Topo I (control for nuclear fraction) and glyceraldehyde 3-phosphate dehydrogenase (GAPDH; control for cytoplasmic fraction). Click here for file

Additional file 4: Figure S4 Showing that enzalutamide (Enza) inhibits androgen-mediated growth of MDA-MB-453 tumors. MDA-MB-453 cells were injected orthotopically in the mammary gland of female NOD-SCID-IL2Rgc^-/-^ mice. Three groups had a DHT pellet implanted subcutaneously and one group had no pellet (Vehicle). Once the tumors reached 100 mm^3^, the mice were given vehicle (Vehicle and DHT groups) or Enza at 10 mg/kg or 25 mg/kg, by daily oral gavage. (A) Tumor volume was measured weekly by caliper. Error bars represent standard error of the mean. *P < 0.05, **P < 0.01, ***P < 0.001 for DHT versus DHT + Enza (10 mg/kg) and DHT + (25 mg/kg), Wilcoxon rank sum. (B) Tumors were excised and weighed at the end of the experiment. ***P < 0.001, analysis of variance with Bonferroni’s multiple comparison test correction. (C) Tumor sections stained for AR. Nuclear AR staining was quantified and representative images (200× magnification) are shown below. *P < 0.05, Kruskal–Wallis with Dunn’s multiple comparison test correction. Click here for file

Additional file 5: Table S1 Presenting the competitive radioligand binding assay with enzalutamide competing with 0.5 nM [3H] estradiol for binding to ERα and ERβ. The competing reference ligand was 1 μM diethylstilbestrol, which gave 50% inhibition at 0.5 nM on ERα and 0.9 nM on ERβ, while enzalutamide at concentrations up to 100 mM only gave between 1 and 4% inhibition on ERα and between 1 and 6% on ERβ. Click here for file

Additional file 6: Figure S5 Showing that enzalutamide (Enza) affects ER protein differently than tamoxifen *in vivo* in MCF7 xenografts. Immunohistochemical staining of ER performed on formalin-fixed paraffin-embedded MCF7 tumor sections (*n* = 8 E2 and E2 + TAM, and *n* = 9 E2 + Enza) scored by pathologist for (A) percent positive nuclear staining (**P < 0.005) and (B) intensity. (C) Overall percent positive signal quantified by ImageJ. *P < 0.05. (D) Representative images at 1,000 ×. Click here for file

Additional file 7: Figure S6 Showing that treatments did not affect mouse body weights in any of the three xenograft experiments. Average mouse weights in grams for (A) mice with MCF7 xenografts in the E2, E2 + enzalutamide (Enza), and E2+ tamoxifen (Tam) treatment groups at the end of the study (day 11); (B) mice with MCF7 xenografts in the DHT versus DHT + Enza treatment groups at the end of the study (day 19); and (C) mice with MDA-MB-453 xenografts treated with vehicle, DHT alone, DHT + 25 mg/kg MDV3100 (Enza), or DHT + 10 mg/kg MDV3100 (Enza) throughout the experiment. Click here for file
