# Supplementary figures and images for: Role of the androgen receptor in breast cancer and preclinical analysis of enzalutamide
Source: Breast Cancer Res. 2014 Jan 22;16(1):R7. doi: 10.1186/bcr3599 (PMC3978822; doi:10.1186/bcr3599)

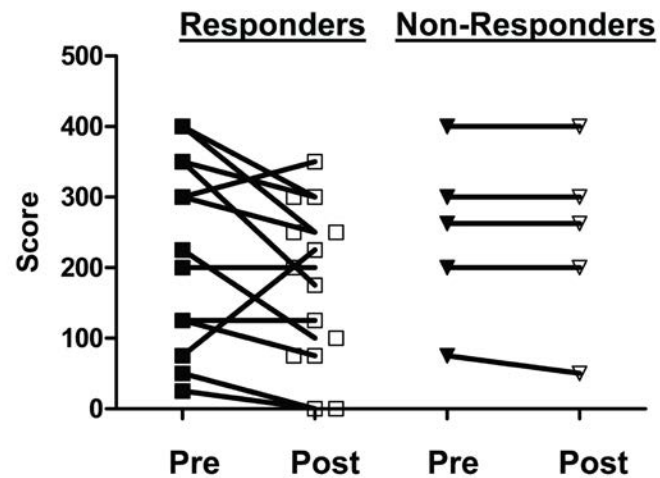

**Responders**

**Non-Responders**

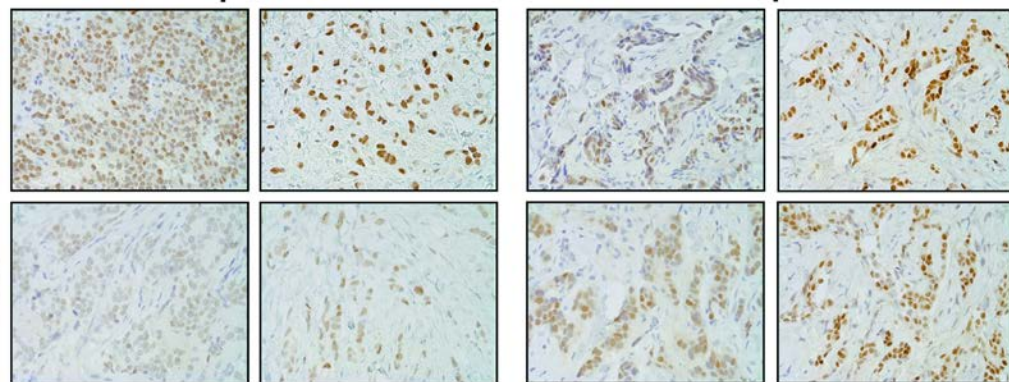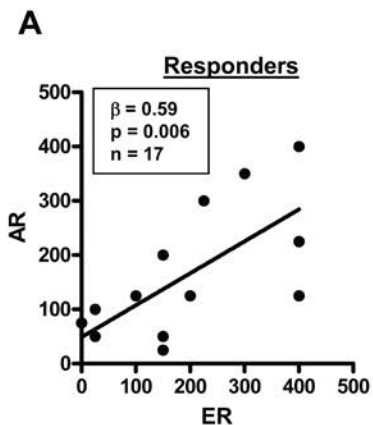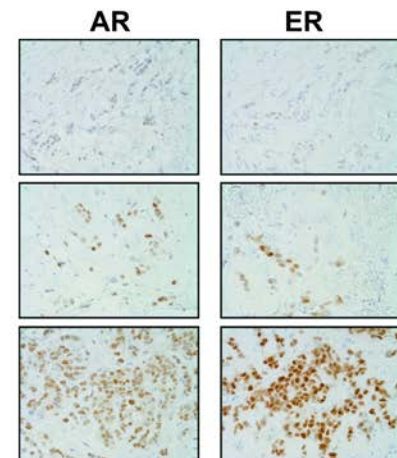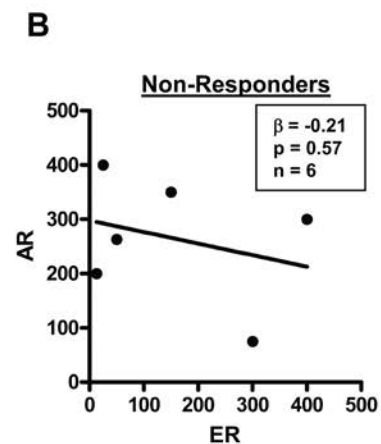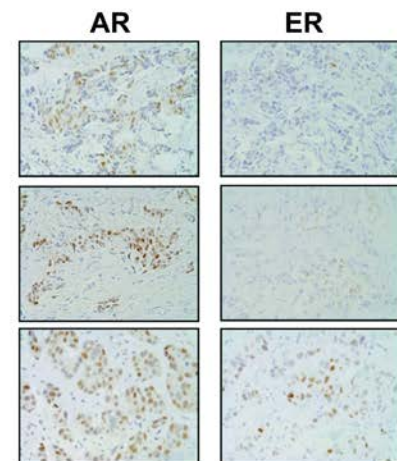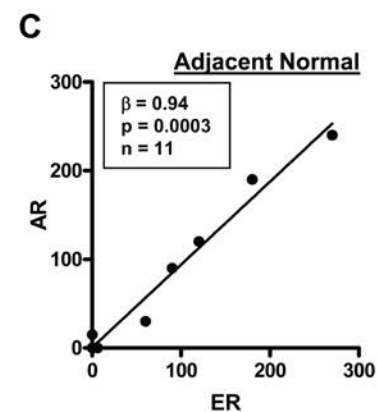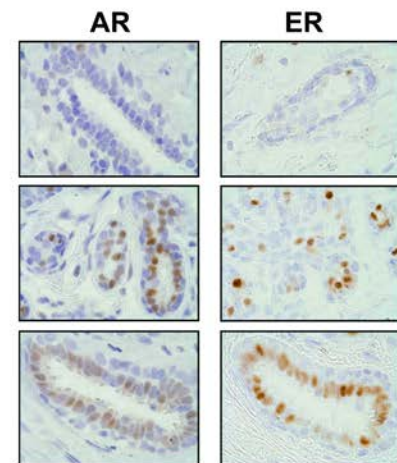

Supplemental Figure 1

Supplement: Additional file 1: Figure S1 — Showing breast tumors that respond to endocrine therapy tend to have decreased AR expression while nonresponders tend to maintain AR expression. There is a positive correlation between AR and ER in responsive tumors and uninvolved adjacent epithelium. Patients received 4 months of neoadjuvant endocrine therapy (exemestane or exemestane + tamoxifen). Core biopsies taken prior to treatment (pre) and a tumor sample at the time of surgery (post) were stained for AR expression. Graph depicts the AR score (percent cells positive for nuclear AR staining versus intensity) in the pre and post treatment samples for those who responded to the endocrine therapy versus nonresponders. P = 0.064, Wilcoxon matched-pair test (left top). Staining of AR in representative responsive and nonresponsive tumors pre versus post treatment is shown below (400× magnification) (left, bottom). In the same tumors, staining score (percent positive staining × intensity) for nuclear AR was plotted on the y axis and ER on the x axis for patients who responded (A, graph) versus those who did not (B, graph). Normal uninvolved glands adjacent to tumors were scored for AR and ER (C, graph). The slope of the line (β) is indicated, as well as the P value, Spearman correlation. Representative images of AR and ER staining (400× magnification) in responders (A, right), nonresponders (B, right) and normal adjacent (C, right) (1,000× magnification). [file bcr3599-S1.pdf]

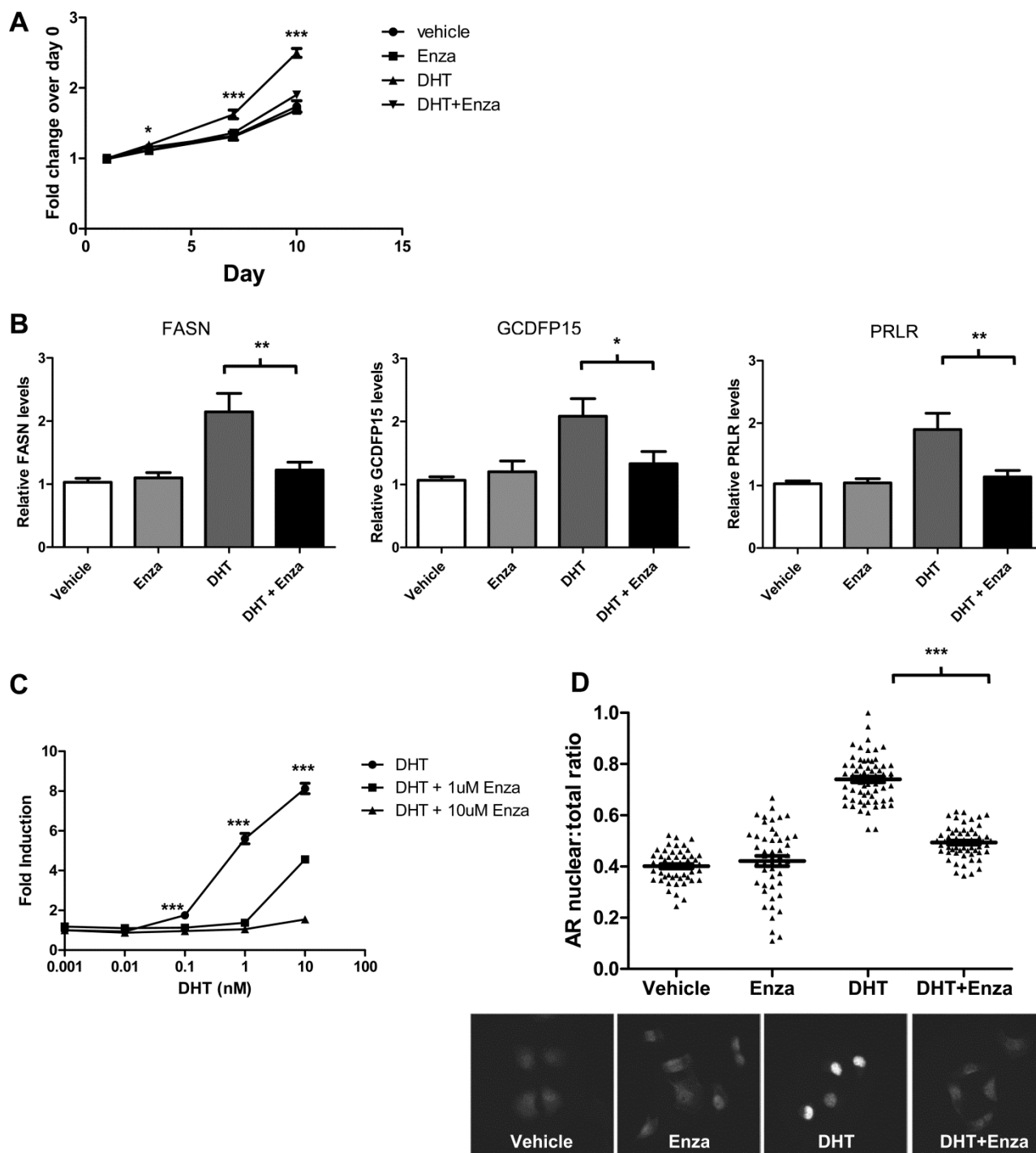

Supplemental Figure 2

Supplement: Additional file 2: Figure S2 — Showing that enzalutamide (Enza) abrogates DHT-mediated proliferation in ER-negative breast cancer cells. (A) MTS proliferation assays were performed in MDA-MB-453 cells treated with vehicle, 10 nM DHT, 10 μM Enza or DHT + Enza. Error bars = standard error of the mean (SEM). (B) Real-time polymerase chain reaction for androgen responsive genes fatty acid synthase (FASN), gross cystic disease fluid protein (GCDFP-15, also called prolactin inducible protein) and prolactin receptor (PRLR) was performed from RNA harvested from MDA-MB-453 breast cancer cells treated with vehicle, 10 μM Enza, 10 nM DHT or DHT + Enza for 24 hours. Genes normalized to 18S and relative to vehicle. *P < 0.05, **P < 0.01 for Student’s t test. (C) MDA-k2b cells, which contain an androgen responsive luciferase construct, were treated for 24 hours with various concentrations of DHT alone or in combination with 1 or 10 μM Enza prior to luciferase assay, and luciferase units relative to the 0.001 nM DHT are shown. Error bars = SEM. (D) MDA-kb2 cells were treated as indicated for 3 hours. Nuclear and total AR staining was quantified with graph indicating the ratio of nuclear to total AR (each triangle represents one cell). Representative images (600× magnification). For proliferation and luciferase assays and the quantification of nuclear/total AR ratio, *P < 0.05, **P < 0.01, ***P < 0.001 for DHT versus DHT + Enza, analysis of variance with Bonferroni’s multiple comparison test correction. [file bcr3599-S2.pdf]

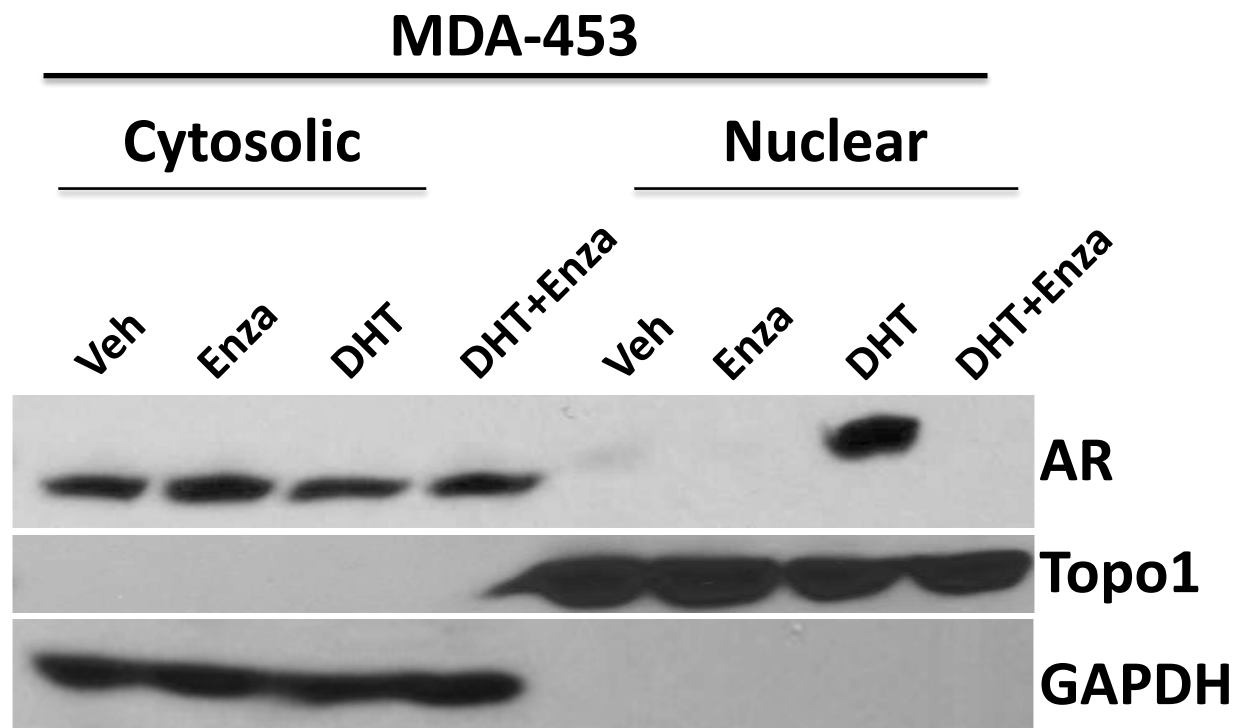

Supplement: Additional file 3: Figure S3 — Showing that enzalutamide (Enza) impairs DHT-mediated nuclear entry of AR in apocrine breast cancer cells. MDA-453 cells were treated with vehicle, 10 nM DHT, 10 μM enzalutamide or DHT + Enza for 3 hours. After nuclear and cytoplasmic fractionation, lysates were immunoblotted for AR, Topo I (control for nuclear fraction) and glyceraldehyde 3-phosphate dehydrogenase (GAPDH; control for cytoplasmic fraction). [file bcr3599-S3.pdf]

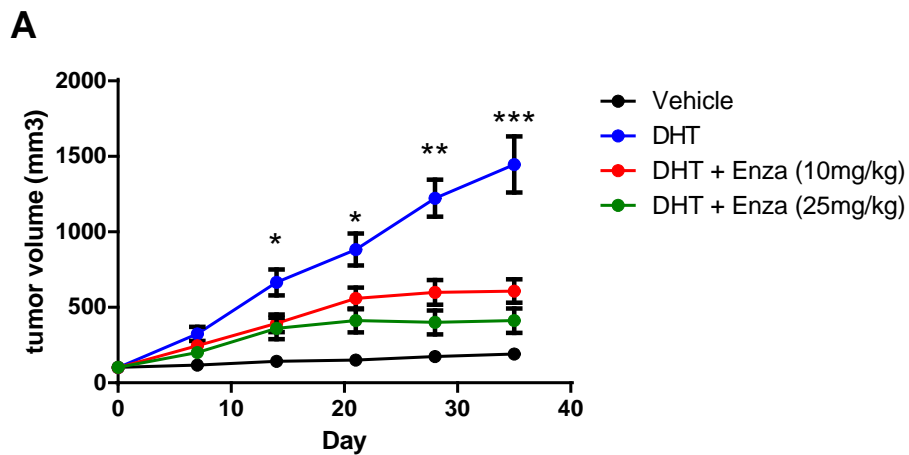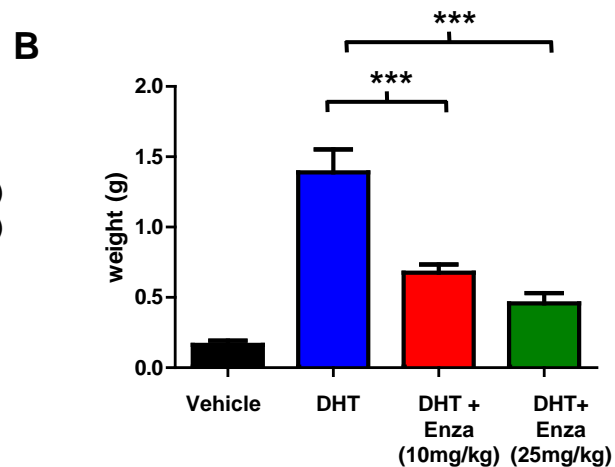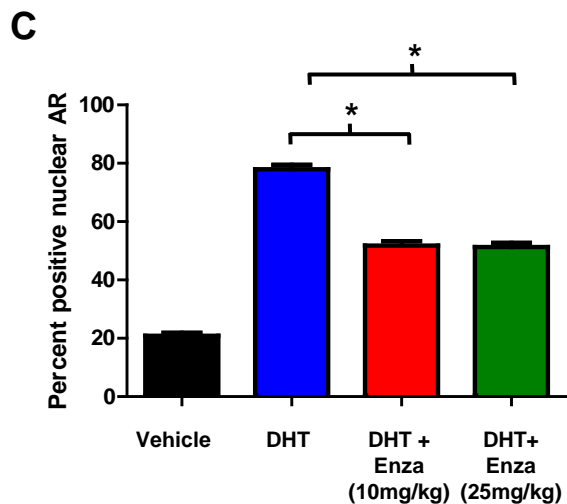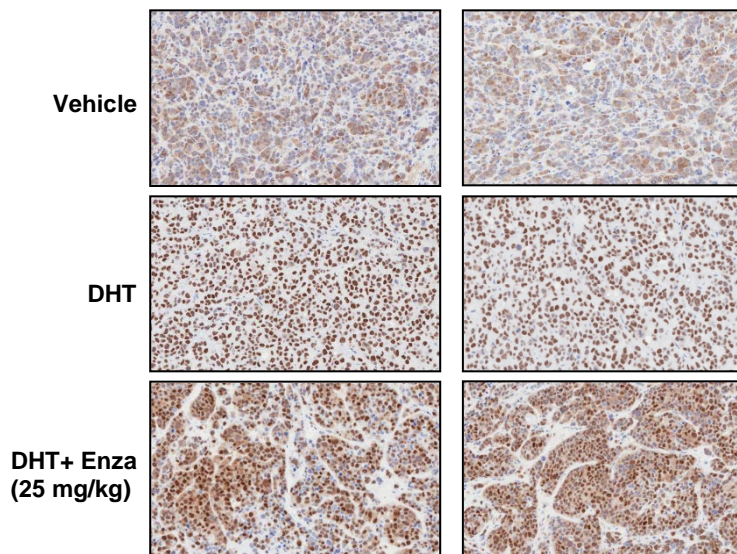

Supplemental Figure 4

Supplement: Additional file 4: Figure S4 — Showing that enzalutamide (Enza) inhibits androgen-mediated growth of MDA-MB-453 tumors. MDA-MB-453 cells were injected orthotopically in the mammary gland of female NOD-SCID-IL2Rgc-/- mice. Three groups had a DHT pellet implanted subcutaneously and one group had no pellet (Vehicle). Once the tumors reached 100 mm3, the mice were given vehicle (Vehicle and DHT groups) or Enza at 10 mg/kg or 25 mg/kg, by daily oral gavage. (A) Tumor volume was measured weekly by caliper. Error bars represent standard error of the mean. *P < 0.05, **P < 0.01, ***P < 0.001 for DHT versus DHT + Enza (10 mg/kg) and DHT + (25 mg/kg), Wilcoxon rank sum. (B) Tumors were excised and weighed at the end of the experiment. ***P < 0.001, analysis of variance with Bonferroni’s multiple comparison test correction. (C) Tumor sections stained for AR. Nuclear AR staining was quantified and representative images (200× magnification) are shown below. *P < 0.05, Kruskal–Wallis with Dunn’s multiple comparison test correction. [file bcr3599-S4.pdf]

**A**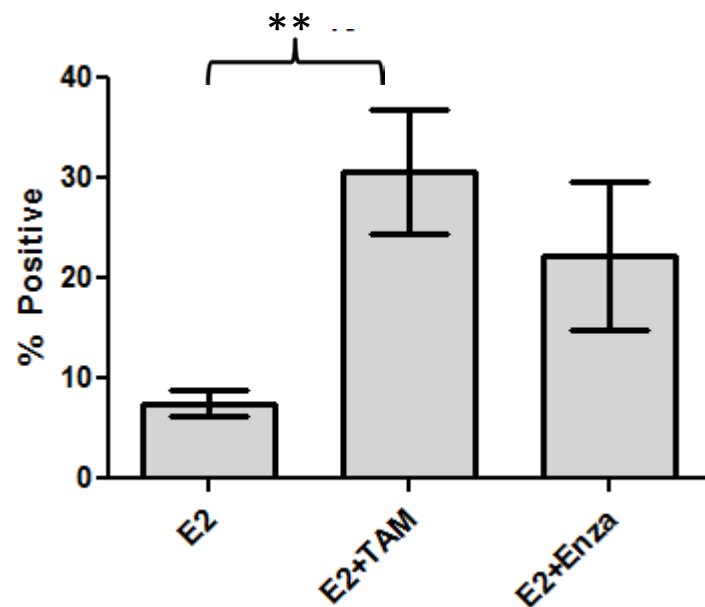**B**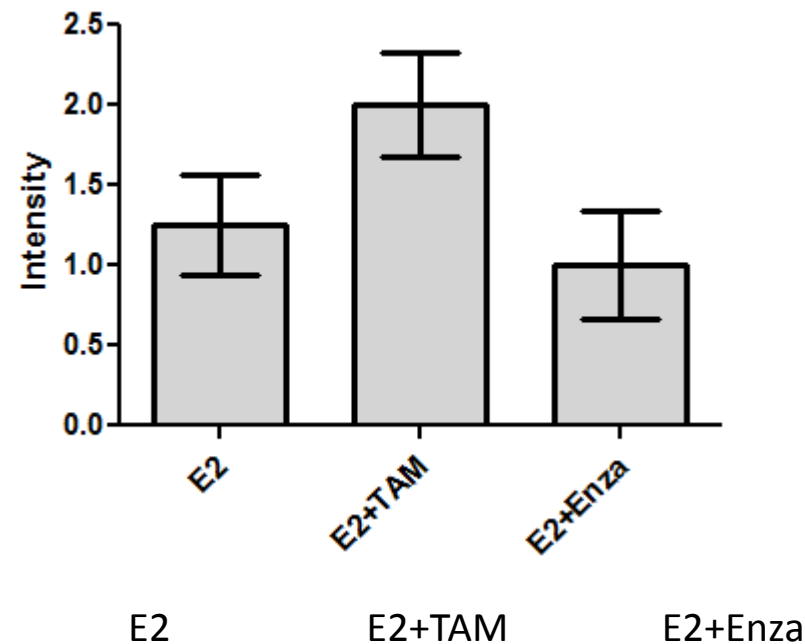**C**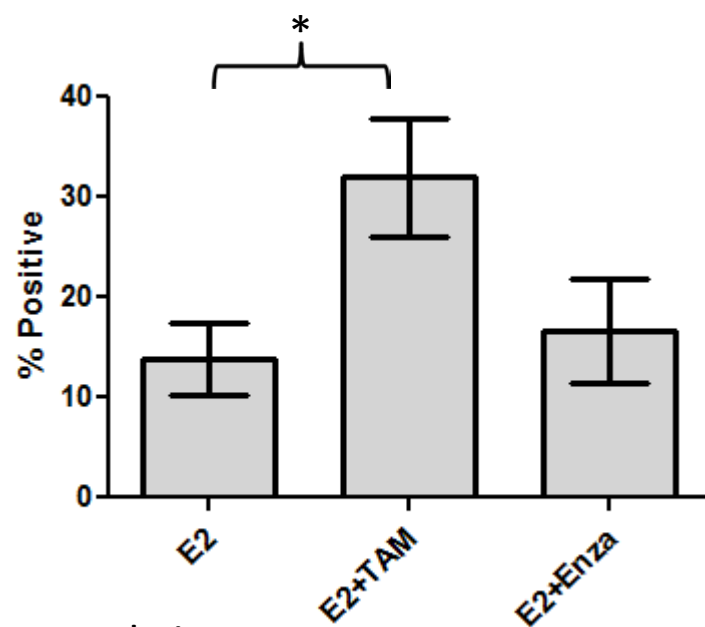**D**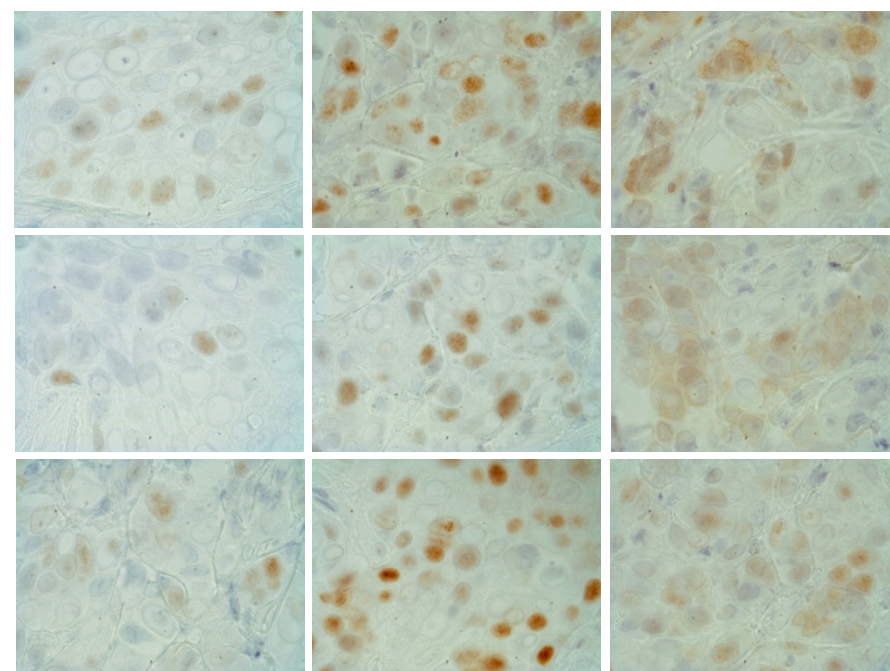

Supplement: Additional file 5: Table S1 — Presenting the competitive radioligand binding assay with enzalutamide competing with 0.5 nM [3H] estradiol for binding to ERα and ERβ. The competing reference ligand was 1 μM diethylstilbestrol, which gave 50% inhibition at 0.5 nM on ERα and 0.9 nM on ERβ, while enzalutamide at concentrations up to 100 mM only gave between 1 and 4% inhibition on ERα and between 1 and 6% on ERβ. [file bcr3599-S5.pdf]

## MCF7 xenograft experiments

A.

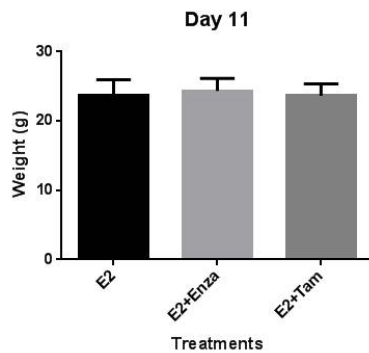

B.

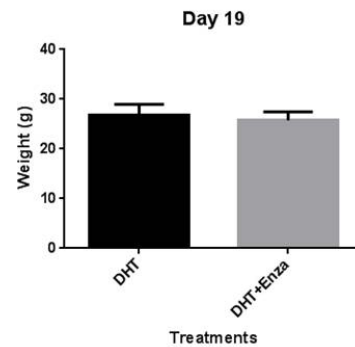

## MDA-MB-453 xenograft experiment

C.

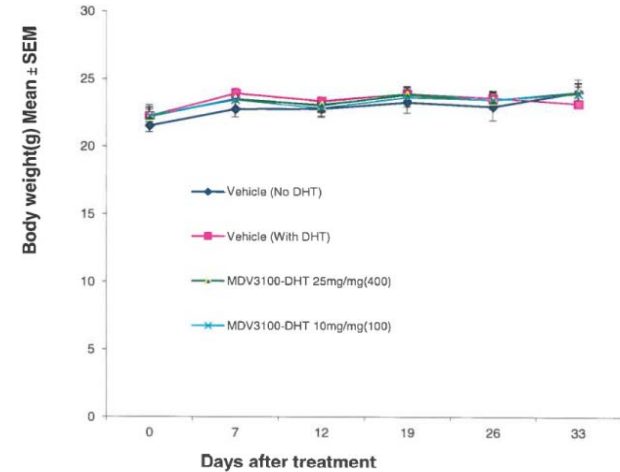

Supplement: Additional file 6: Figure S5 — Showing that enzalutamide (Enza) affects ER protein differently than tamoxifen in vivo in MCF7 xenografts. Immunohistochemical staining of ER performed on formalin-fixed paraffin-embedded MCF7 tumor sections (n = 8 E2 and E2 + TAM, and n = 9 E2 + Enza) scored by pathologist for (A) percent positive nuclear staining (**P < 0.005) and (B) intensity. (C) Overall percent positive signal quantified by ImageJ. *P < 0.05. (D) Representative images at 1,000 ×. [file bcr3599-S6.pdf]
