# Supplementary material for: Role of the androgen receptor in breast cancer and preclinical analysis of enzalutamide
Source: Breast Cancer Res. 2014 Jan 22;16(1):R7. doi: 10.1186/bcr3599 (PMC3978822; doi:10.1186/bcr3599)
Supplement: Additional file 7: Figure S6 — Showing that treatments did not affect mouse body weights in any of the three xenograft experiments. Average mouse weights in grams for (A) mice with MCF7 xenografts in the E2, E2 + enzalutamide (Enza), and E2+ tamoxifen (Tam) treatment groups at the end of the study (day 11); (B) mice with MCF7 xenografts in the DHT versus DHT + Enza treatment groups at the end of the study (day 19); and (C) mice with MDA-MB-453 xenografts treated with vehicle, DHT alone, DHT + 25 mg/kg MDV3100 (Enza), or DHT + 10 mg/kg MDV3100 (Enza) throughout the experiment. [file bcr3599-S7.doc]

**Additional file 7: Table S1. Competitive binding assay with 0.5 nM [3H] estradiol.**

|  | **[Enza]** | **% Inh.** | **IC50** |
| --- | --- | --- | --- |
| ERa | 100 mM | 1 | >100 mM |
|  | 10 mM | 4 |  |
|  | 1 mM | -2 |  |
|  | 0.1 mM | 3 |  |
| ERb | 100 mM | 3 | >100 mM |
|  | 10 mM | 6 |  |
|  | 1 mM | 1 |  |
|  | 0.1 mM | -1 |  |

Competing reference ligand was 1 μM Diethylstilbestrol,which gave 50% inhibition at 0.5 nM

on ERa and 0.9nM on ERb
